# Influence of the Electrode Deposition Method of Graphene-Based Catalyst Inks for ADEFC on Performance

**DOI:** 10.1021/acsami.3c09192

**Published:** 2023-08-17

**Authors:** Michaela Roschger, Sigrid Wolf, Richard Hasso, Boštjan Genorio, Selestina Gorgieva, Viktor Hacker

**Affiliations:** † Institute of Chemical Engineering and Environmental Technology, Graz University of Technology, Inffeldgasse 25/C, 8010 Graz, Austria; ‡ Faculty of Chemistry and Chemical Technology, University of Ljubljana, Večna pot 113, 1000 Ljubljana, Slovenia; § Faculty of Mechanical Engineering, University of Maribor, Smetanova ulica 17, 2000 Maribor, Slovenia

**Keywords:** alkaline direct ethanol fuel cell, restacking graphene sheets, half-cell GDE measurements, N rGO based electrodes, durability

## Abstract

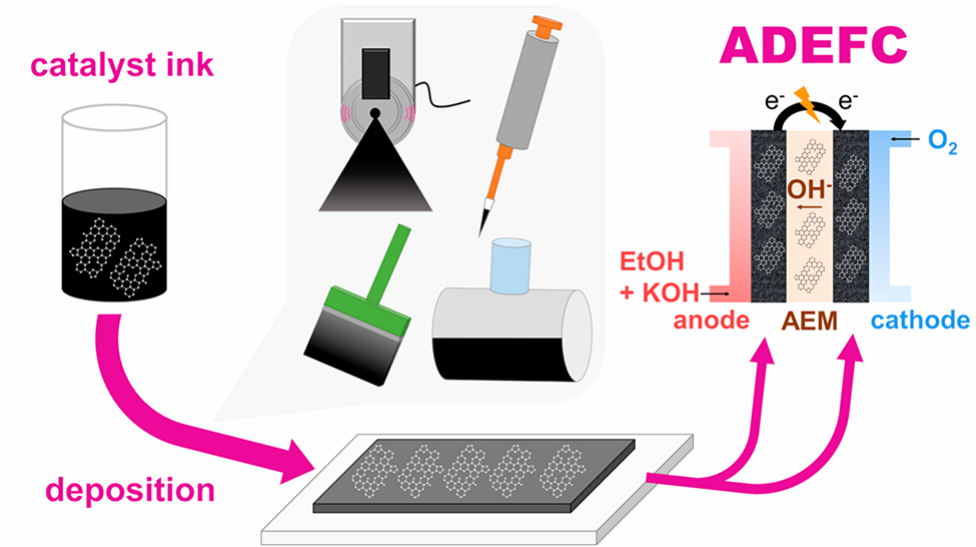

The utilization of graphene as a catalyst support has garnered significant attention due to its potential for enhancing fuel cell performance. However, a critical challenge in electrode production still lies in the electrode preparation technologies and the restacking of graphene sheets, which can greatly impact the fuel cell performance alongside with catalyst development. This study aimed to investigate the impact of different electrode deposition methods for N-rGO-based catalyst inks on catalyst layer morphology, with a specific focus on graphene sheet orientation and its influence on the performance of alkaline direct ethanol fuel cells (ADEFCs). The dispersion behavior and ink stability of the catalysts were assessed using ultraviolet–visible light (UV-vis), ζ potential, and dynamic light scattering techniques. The morphology and physical properties of the gas diffusion electrodes (GDEs) were analyzed through Brunauer–Emmett–Teller measurements, contact angle measurements and scanning electron microscopy (SEM) combined with energy-dispersive spectroscopy. The electrochemical behavior was evaluated both ex-situ, utilizing half-cell GDE measurements, and in situ, through single-cell tests. The N-rGO-based membrane electrode assembly, comprising Pt-free catalysts and a biobased membrane, exhibited outstanding performance in ADEFCs, as evidenced by high maximum power density values and long-term durability. The N-rGO-based membrane electrode assembly has demonstrated remarkable potential for high-performance fuel cells, presenting an exciting avenue for further exploration.

## 1 Introduction

Graphene, a graphite derivative, is a two-dimensional (2D) nanosheet layer of carbon atoms that are *sp*^2^ hybridized and arranged in a honeycomb structure. Since its discovery in 2004 and the awarding of the Nobel Prize in 2010 to Andre Geim and Konstantin Novoselov for their pioneering work on graphene, research on the synthesis, the chemical modification, and the application of this fascinating material has increased significantly.^1−7^ This rise in interest can be attributed to the unique properties of graphene.^3,4,6−9^ There are different synthesis pathways for graphene available, whereby the Hummers method (chemical oxidation of graphene) is the most popular chemical process and results in graphene oxide (GO), which has oxygen containing functional groups and defects. These modifications change the properties of the material, from a hydrophobic surface to a hydrophilic one and from an intrinsically conductive material to an insulating one (*sp*^3^ hybridization). The chemical or thermal reduction of the attached oxygen functional groups from GO results in reduced graphene oxide (rGO) and the restoration of parts of the *sp*^2^ structure and, thus, conductivity.^1,3,4,7−10^ The modification of the carbon backbone of GO by doping with heteroatoms, such as nitrogen (e.g., NGO or N-rGO), can further increase the conductivity.^5,8,11^

The excellent properties of graphene make it particularly interesting for the use in sustainable electrochemical devices such as fuel cells, super capacitors, and photovoltaics.^1−4^ The incorporation of graphene compounds in different components of fuel cells (catalysts, membranes, and bipolar plates) increases their lifetime and durability, as well as the performance.^1,4,5,7−9^ The addition of graphene to polymeric fuel cell membranes enhances the ionic conductivity, reduces the fuel permeability, increases tensile strength and improves the water retention, as already discussed in several review articles.^1,4,5,8^ The main focus, however, is on the development of graphene-based catalysts (e.g., the support material for metal nanoparticles) and electrodes since there is not only an increase in electronic conductivity but also a significant increase in the effective surface area and thus activity.^1,5,7,8^

Extensive research is currently being conducted on the utilization of graphene compounds in the anodic catalysts,^12−,25^ cathodic catalysts,^26−34^ and membranes^35−38^ for use in alkaline direct ethanol fuel cells (ADEFCs). The ADEFC has emerged as a significant type of fuel cell, garnering increasing importance in recent years. Notably, ADEFCs offer a range of compelling advantages, including their environmental friendliness, sustainability, and their use of liquid fuel, which facilitates convenient transport and handling. However, further development for all the components of the membrane electrode assembly (MEA) is needed: (i) the complete oxidation of ethanol at the anode catalysts (e.g., Pt or Pd) and extended durability, (ii) enhancement of the long-term stability and ionic conductivity of anion exchange membranes (AEMs), and (iii) improvement of the ethanol tolerance and activity of the cathode catalysts (e.g., Pt, Ag, Au, manganese oxides, spinels). The theoretical complete electrochemical cleavage of ethanol with OH^–^ (produced at the cathode and transported through the membrane) would result in 12e^–^ and CO_2_. However, complete electrochemical C–C bond splitting is not achieved and a product mixture of acetaldehyde (2e^–^), acetic acid/acetate (4e^–^), and CO_2_/carbonate is formed in the alkaline medium. Therefore, to facilitate the ethanol oxidation reaction and increase the conductivity of the AEMs, alkali (OH^–^) is added to the anodic fuel.^39−42^ To overcome these challenges, the research on the introduction of graphene-based compounds was performed and resulted in lower ethanol permeability, higher conductivity, and thermal stability for the AEMs.^35,36,38^ The implementation in the anode catalysts and cathode catalysts as a support material results in improved electron transport and reduced agglomeration of the nanoparticles and thus higher activity.^13,15,22,26,29,30^

Beside the research on membranes and catalysts, which is mostly investigated for graphene-based catalysts at the fundamental level by deposition on a glassy carbon substrate using rotating disk experiments,^3,7,8,19^ the electrode fabrication or MEA production is another crucial aspect that demands careful consideration. Several options exist for catalyst-powder based production, each of which can impact the stability and reproducibility of the final product.^43^ The fabrication methods for graphene-based catalysts in solutions, e.g., isopropanol and water, for different fuel cells include spraying, doctor blading, brushing, painting, pasting, and spin coating.^13,18,23,27,28,30,44−49^ The restacking of graphene sheets by π–π interactions and strong van der Waals attraction and thus, the aggregation of the catalyst material in solutions during electrode production represents a challenge, as it lowers the overall performance. The reason for the complexity of producing stable suspensions can be attributed to the different hydrophobicity of the various graphene compounds and, therefore, their dissolving behavior.^2,8−10,50−55^

The aim of this work was thus to study the optimal electrode deposition method of N-rGO supported anodic (PdNiBi-based) and cathodic (Ag–Mn_*x*_O_*y*_-based) catalyst inks. These utilized reactive catalysts have been previously studied in the literature,^25,29,56^ but have never been used on the N-rGO support material as electrode material in ADEFC. Hence, the dispersion behavior and the stability of these catalysts in distinct solvent ratio mixtures was evaluated. The morphology and distribution of the prepared electrodes was analyzed and the activity was determined with half-cell gas diffusion electrode (GDE) measurements. The best-performing electrodes were measured with single cell tests at different operating conditions. In accordance with the best of our knowledge, the first completely N-rGO based MEA (both catalysts and membrane) for use in ADEFCs was thus produced. In addition, the durability of the MEA was examined over time. The N-rGO-based MEA reached higher power densities and a higher long-term durability, compared to commercially available materials.

## 2 Experimental Section

In this study, N-rGO supported anodic and cathodic catalysts were synthesized and the dispersion behavior of these catalysts in various solvent mixtures and the stability of the resulting catalyst inks was evaluated. The effects of four different electrode deposition methods of the most stable and optimally dispersed catalyst ink for each catalyst on the morphology, activity and performance in an ADEFC were investigated.

### 2.1 Chemicals and Materials

Ultrapure water (∼18 MΩ cm, Barnstead NANOpure-Water Purification system, Dubuque, IA, USA) was used for all productions of solutions. For the synthesis of the N-rGO supported cathodic and anodic catalysts, the following chemicals were used. The support material NrGO (N-htGO (KS44)) was prepared as reported in a previous study.^57^ Silver nitrate (AgNO_3_, ≥99.8% p.a.) and potassium permanganate (KMnO_4_, ≥99.0% p.a.) were delivered by Merck (Darmstadt, Germany). Palladium chloride (PdCl_2_, anhydrous, 59%–60% Pd basis), bismuth(III) chloride (BiCl_3_, reagent grade, ≥98%) and nickel(II) nitrate hexahydrate (Ni(NO_3_)_2_·6H_2_O, 99% trace metal basis) were purchased from Sigma–Aldrich (Darmstadt, Germany). Hydrochloric acid (HCl, ROTIPURAN 37% fuming, p.a., ACS, ISO) and 2-propanol (99.9% p.a.) were supplied by Carl Roth (Karlsruhe, Germany). Sodium hydroxide (NaOH, ≥98%, ACS, pellets) and sodium borohydride (NaBH_4_, 97% purity) were delivered by Honeywell Fluka (Charlotte, NC, USA) and Alfa Aesar (Haverhill, MA, USA), respectively.

For the preparation of the catalyst inks, 2-propanol (99.9% p.a., Carl Roth, Karlsruhe, Germany) and Nafion Solution (NS-5, PFSA 5%, Quintech, Göppingen, Germany) were utilized.

The following materials and chemicals were used for the electrochemical and single-cell measurements. Ethanol (EtOH, 99.9% p.a.) and KOH (≥85%, p.a., pellets) were derived from Carl Roth (Karlsruhe, Germany). Carbon paper (Sigracet 29 BC, 0.235 mm thick), carbon cloth (ELAT - Hydrophilic Plain Cloth, 0.406 mm thick) and a commercial Pd/C catalyst (40 wt %) were delivered by Fuel Cell Store (College Station, TX, USA). Fumasep FAA-3–50 (anion-exchange membrane, nonreinforced) and a commercial Pt/C catalyst (platinum, nominally 40% on carbon black) were purchased from Fumatech (Bietigheim-Bissingen, Germany) and Alfa Aesar (Haverhill, MA, USA), respectively. A laboratory-made anion exchange N-doped graphene derivative based chitosan-membrane (CS/N-rGONRs) from a previous study was employed.^38^

### 2.2 Catalyst Synthesis

The cathodic N-rGO based catalyst was synthesized as described in previously published literature.^29^ Therefore, the synthesis itself is not described here. The catalyst consists of 30 wt % active material (10 wt % Ag and 20 wt % Mn_*x*_O_*y*_) on the support material N-rGO.

The anodic N-rGO catalyst also consists of 30 wt % active material (85 at. % Pd, 10 at. % Ni, and 5 at. % Bi) on the same support material (N-rGO). This 30 wt % catalyst was prepared as stated in our previous works,^25,56^ with the exception of N-rGO, which was used as support material instead of Vulcan or rGO.

### 2.3 Preparation and Physical Investigation of the Catalyst Inks

The preparation of stable and well-dispersed graphene catalyst suspensions is an important issue for the fabrication of electrodes.^50^ Therefore, different solvent mixtures of water and isopropanol for both catalysts (4 mg mL^–1^) were produced with different relations (*x*:*y*) between isopropanol (*x*) and water (*y*): sample 1 (10:0), sample 2 (9:1), sample 3 (8:2), sample 4 (7:3), sample 5 (6:4), sample 6 (5:5), sample 7 (4:6), sample 8 (3:7), sample 9 (2:8), sample 10 (1:9), and sample 11 (0:10). The amount of Nafion in all solutions was 30 wt % related to the total catalyst weight. The resulting catalyst inks were analyzed after ultrasonication in an ultrasonic bath for 30 min with ultraviolet–visible spectroscopy (UV-vis), with regard to solubility and stability; furthermore, the catalyst inks were analyzed with zeta potential (ζ) measurements to evaluate the aggregation behavior of the particles in the solution and with dynamic light scattering (DLS) to determine the hydrodynamic radius.

The UV-vis measurements were performed with diluted inks (1:100 sample:water) in a quartz glass cuvette with a Shimadzu UV-1800 visible scanning spectrophotometer (Shimadzu, Kyoto, Japan) in a wavelength range between 200 and 800 nm at room temperature (RT) at time intervals of 0, 1, 2, 3, and 6 h. A Litesizer 500 (Anton Paar, Graz, Austria) was used for the ζ potential and DLS measurements with diluted inks (1:100 sample:water), which were directly taken after ultrasonication. The measurements were performed at RT with a scattering angle of 175° in an Omega cuvette (Anton Paar, Graz, Austria). Average values and standard deviations from three individual measurements were calculated for all measurements (UV-vis and ζ).

### 2.4 Electrode Fabrication

**Figure 1:**
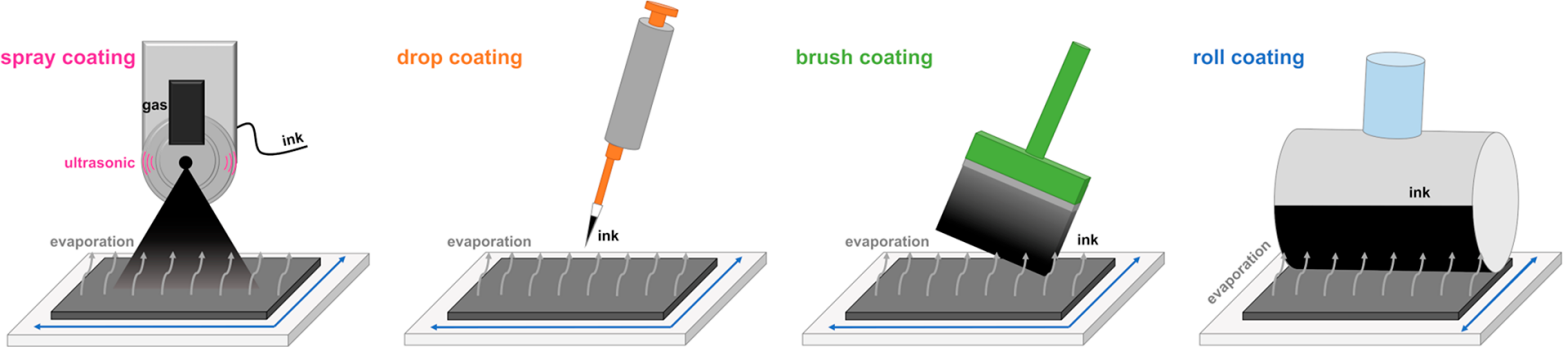
Schematic illustration of the four different electrode deposition methods: ultrasonic spray coating, drop coating, brush coating, and roll coating.

For the determination of the effects of the electrode deposition method of the N-rGO-based catalysts inks on the GDL (carbon cloth comprised the anode and carbon paper comprised the cathode), four different methods (ultrasonic spray coating, drop coating, brush coating, and roll coating) were tested, as shown in Figure 1.

The catalyst ink composition (ratio of the solvents) for all tested deposition methods was the same for each catalyst and was selected according to the UV-vis, ζ, and DLS studies. However, the catalyst content for the roll coating method was 40 mg mL^–1^, since, in this production method, a paste rather than an ink was needed.^58^ An active area of 4 cm^2^ of the GDLs, attached to a porous PTFE substrate heated to 80 °C, was coated to produce the GDEs. Care was taken to ensure that the catalyst ink was applied evenly (in multiple directions) and slowly so that the solvent could evaporate. An ultrasonic Sonotech ExactaCoat OP3 spray coater (SonoTek Corporation, USA) with a 120 kHz nozzle was employed for the spray-coating process, along with a pipet for the drop-coating process, a paint brush for the brush-coating process, and a metal roller with handle for the roll-coating process. For all cathodic electrodes, an active material loading of 0.25 mg cm^–2^ was achieved, whereas an active material loading of 0.5 mg cm^–2^ for all anodic electrodes was achieved.

### 2.5 Physicochemical and Electrochemical Electrode Characterization

The prepared electrodes (cathodes and anodes) were physicochemically analyzed with contact-angle measurements, the Brunauer–Emmett–Teller (BET) method and scanning electron microscopy (SEM), and their electrochemical behavior was determined with half-cell GDE experiments and single-cell tests.

SEM analysis coupled with energy-dispersive spectroscopy (EDX) was performed to determine the electrode coating and distribution, as well as the cross section with an FEI-XL20 (Philips, Amsterdam, The Netherlands) and an EDX-detector from remX GmbH (Bruchsal, Germany). Therefore, the electrodes were cut with a sharp scalpel and adhered to the sample holder with conductive carbon tape. The water contact angles (hydrophilicity) of the electrodes were measured with a goniometer (Ossila, South Yorkshire, United Kingdom) by dropping ∼3.5 μL on the surface. The analysis of the contact angles was performed with the Ossila Contact Angle software. The measurements were performed three times at RT and the average values and standard deviations calculated. The BET surface area was determined on an ASAP 2020 Micromeritics (Norcross, GA, USA) instrument by single point analysis after degassing the electrodes (1 cm^2^) in a glass tube under vacuum.

The prepared GDEs (anodes and cathodes) were punched out in circular form (active area: 1 cm^2^) and investigated by half-cell GDE measurements with respect to their electrochemical behavior, such as the oxidation and reduction processes of the active species (and electrochemical surface area (ECSA)) as well as the activity, with respect to ORR (cathodes) and EOR (anodes). The electrodes were labeled according to the method of manufacture and whether they were cathode or anode (with subscripts C or A). A measurement protocol from our previous work^42^ with a Zahner IM6ex potentiostat coupled with a PP240 power-potentiostat (Zahner-elektrik GmbH & Co. KG, Kronach-Gundelsdorf, Germany) was applied and is therefore only briefly described here. The GDEs were measured in a Diskfix electrode holder from Bank Elektronik–Intelligent Controls GmbH (working electrode) in a three-electrode setup in 5 M KOH. A reversible hydrogen electrode (RHE) in a Luggin capillary from Hydroflex gaskatel was used as a reference electrode and a platinized titanium rod from Bank Elektronik–Intelligent Controls GmbH was used as a counter electrode. The cyclic voltammograms (CVs) were conducted after 1 h N_2_-purging and cleaning cycles with a scan rate of 10 mV s^–1^ in a potential range of 0.1–1.0 V vs RHE with the cathodes and in a potential range of 0.05–1.50 V vs RHE and 0.05–1.20 V vs RHE (ECSA determination) with the anodes. The ORR and EOR activity was evaluated with polarization curves, which were post *iR*-compensated (with resistance determination at each measurement point), by supplying either oxygen with a flow rate of 25 mL min^–1^ or a mixture of 5 M KOH and 3 M EtOH with a flow rate of 5 mL min^–1^ through the electrode at RT (condition I), 60 °C (condition II) or 80 °C (condition III).

The best-performing electrode, both cathode and anode in the half-cell GDE tests, was selected for the MEA, and the MEA was designated as the N-rGO based MEA. The MEA for the single cell tests was prepared by assembling the anodic GDE, the pretreated (24 h in 1 M KOH) CS/N-rGONRs AEM and the cathodic GDE together. The fuel cell tests were performed galvanostatically using the same potentiostat as described before and employed a homemade ADEFC test rig (test station) and cell, as described in detail in ref (59). A mixture of 3 M EtOH and 5 M KOH at a flow rate of 5 mL min^–1^ was used as fuel at the anode while oxygen at a flow rate of 25 mL min^–1^ was used at the cathode. Three different operating conditions were tested: condition I (RT, pure O_2_), condition II (60 °C, humidified O_2_), and condition III (80 °C, humidified O_2_). Furthermore, constant-current discharging tests (durability tests) were conducted by applying 15 mA cm^–2^ for 12 h at condition III and by measuring the voltage loss.^60^

In addition, an MEA of commercial materials: a Pd/C catalyst at the anode, Fumasep FAA-3-50 anion exchange membrane and a Pt/C catalyst at the cathode (denoted as comm. MEA) with the same active material loadings was fabricated and tested for comparison.

## 3 Results and Discussion

### 3.1 Physical Investigation of the Catalyst Inks

**Figure 2:**
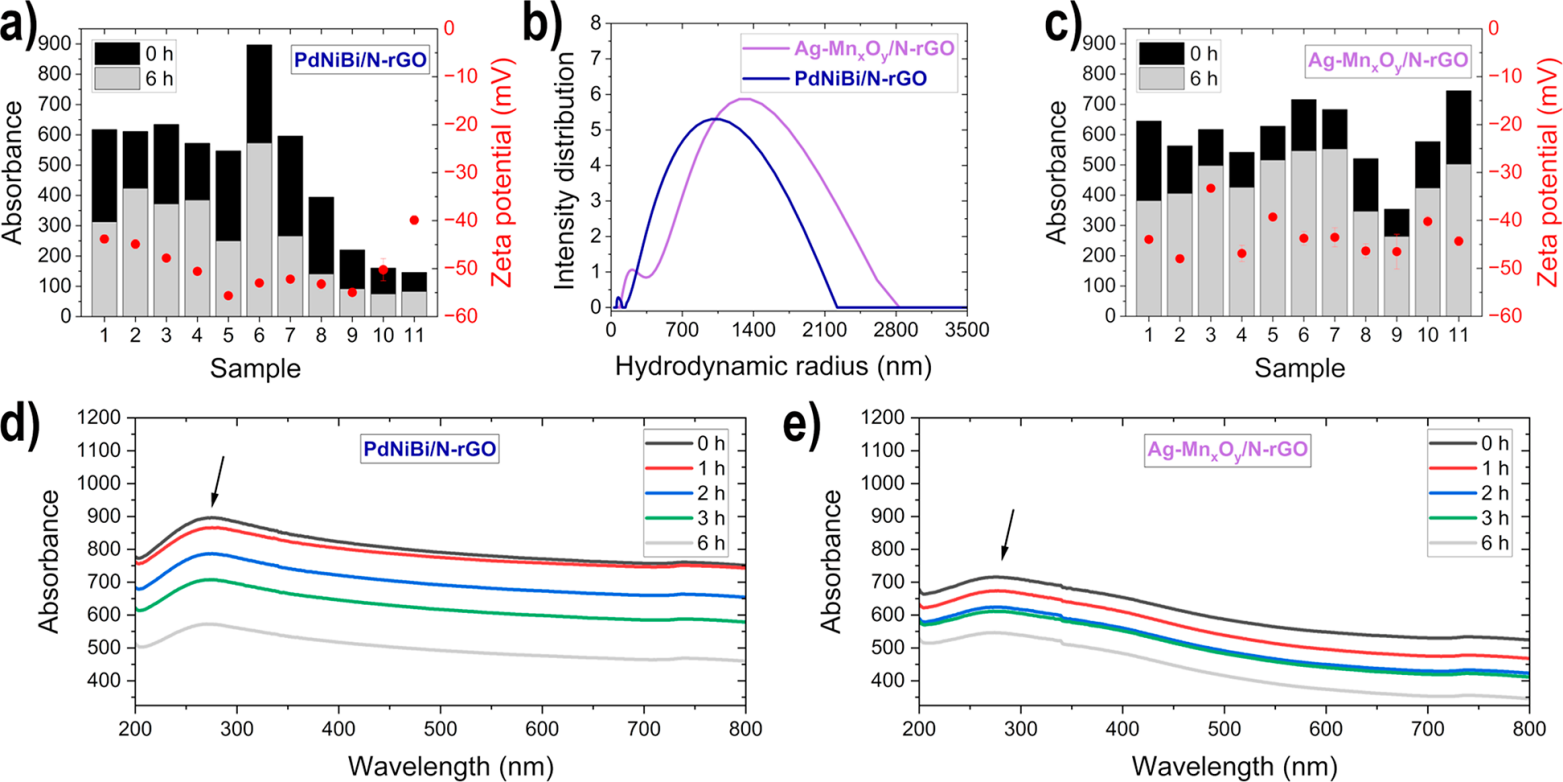
Physical investigation results of the catalyst inks; Zeta potential and UV–vis absorbance for (a) the PdNiBi/N-rGO and (c) the Ag-Mn*_x_*O*_y_*/N-rGO catalyst inks; (b) hydrodynamic radius determined with DLS (both sample 6); and catalyst ink stability determination (0, 1, 2, 3, 6 h) and UV-vis spectra (both sample 6) for (d) PdNiBi/N-rGO and (e) Ag-Mn*_x_*O*_y_*/N-rGO ink.

The stability and agglomeration of the catalyst inks affect the behavior of the electrode and its fabrication.^50,52^ The aggregation size of the ionomer and the catalyst particles, and thus the physical and mass transfer properties of the catalyst layer, are controlled by the nature of the dispersion medium.^58^ Therefore, the absorbance of the catalyst particles, and thus solubility as well as stability, was determined with UV-vis spectroscopy in various solvent mixtures (isopropanol and water). Moreover, the stability or aggregation behavior was analyzed with ζ potential measurements in combination with DLS for the evaluation of the hydrodynamic radius. In Figure 2 and Table S1 and S2 in the Supporting Information, the physical investigation results for the different catalyst ink solutions can be found.

The maximum absorption (marked with the arrow in Figures 2d and 2e) of the catalyst inks was used to determine the solubility of the catalysts in the solution medium because a higher absorption intensity is the result of a higher turbidity caused by the catalyst. Moreover, Poorsargol et al.^53^ used the absorption intensity to evaluate the concentration of the graphene dispersions. The peak maximum for both N-rGO based catalyst inks is located at ∼280 nm and can be attributed to the *sp*^2^ hybridized C=C bonds.^55^ The content of the attached oxygen groups on the N-rGO is relatively low since, as Liu et al.^55^ demonstrated, that the absorption spectra for GO demonstrates a maxima for the aromatic C–C bonds (π–π* transition) at 238 nm, which red-shifts with the reduction of oxygen groups, due to conversion from *sp*^3^ to *sp*^2^ coordinated carbon atoms and a small peak at ∼303 nm for the C=O bonds (n-π*), which disappears with reduction. The freshly prepared PdNiBi/N-rGO inks (Figure 2 a–black bars) indicate a constant absorption maximum for the samples 1–5, a very high value for sample 6, and subsequently, for samples 7–11, a very sharp rapid decline. The low oxygen content in the N-rGO sheets, as seen with the position of the peak maxima, causes an increase in the π–π restacking forces of the carbon atoms and thus an instability in water.^55^ The absorption maxima, however, is in the same area for all samples for the Ag–Mn_*x*_O_*y*_/N-rGO inks (Figure 2c black bars). Consequently, they show similar solubility behavior, regardless of the composition of the varying solvent ratio. We consider that the different active material on the N-rGO sheets, which contains transition-metal oxide, is the reason for this. Transition-metal oxides on the surface of the N-rGO sheets are able to minimize restacking, and therefore no substantial change can be seen.^61^ Moreover, the oxygen content or the quantity of polar functionalities has an influence on the hydrophilicity of the catalyst.^55^

The average hydrodynamic radius (Figure 2b and Tables S1 and S2), which describes the particle with solvation shell and was determined with DLS of all the samples, is relatively large (700–1900 nm), due to the large size of the N-rGO sheets (graphene sheets are typically in the low micromolal range, as can be seen on electron microscopy images in the literature^25,26,45^) and the Nafion ionomer in the solution. The values are basically higher for the samples containing greater amounts of water due to the aggregation of the N-rGO sheets (van der Waals forces and π–π stacking),^55^ and we assume that the hydrophilic character of Nafion and, thus, its attraction to water also has an impact. In addition, the dielectric constant of the different solvent mixtures decreases with higher quantity of isopropanol in the solution,^62^ which, in turn, influences the dispersion behavior of the ionomer (distribution and dimensions of aggregates) and of the catalyst.^63^

The stability of dispersions can be easily described with the ζ potential, as it specifies the electrostatic repulsion between particles, whereas values of < −25 mV or >25 mV are expected to be stable.^53^ The stability of graphene-compounds in solution depends on the balance between electrostatic repulsion and van der Waals attraction.^54^ The measured ζ potential values are more negative than −25 mV for all measured samples and are therefore considered as stable. The values are negative because the Nafion ionomer or its ionic properties interact with the N-rGO sheets and transfer an effective charge onto them.^53^ For the PdNiBi/N-rGO samples (Figure 2a), a clear trend can be seen: the ζ potential becomes more and more negative starting from sample 1 (decreasing isopropanol content), has its lowest value at approximately samples 5 and 6 and then becomes less negative again with higher water content. The higher water content, as described before, enables the π–π restacking of the N-rGO sheets.^55^ The first decrease (higher potential values) may refer to the Nafion content (hydrophilic) in the solution. For the Ag–Mn_*x*_O_*y*_/N-rGO samples (Figure 2 c), no clear upward or downward trend is observed (all samples are in a similar range), as shown previously with the UV–vis measurements, which can again be attributed to the different active material on the N-rGO sheets.^61^

The stability of all tested catalyst inks over time is not particularly high, as the absorption maxima in Figures 2d and 2e and Tables S1 and S2 in the Supporting Information decrease over the measured time period due to aggregation of the N-rGO sheets.^50^ However, sample 6 still show the highest absorption values after 6 h (gray bars in Figures 2a and 2c) and thus still the highest catalyst content in solution and the least sedimentation. The PdNiBi/N-rGO samples show a higher decrease in the absorption maxima in 6 h in comparison to the Ag–Mn_*x*_O_*y*_/N-rGO samples, due to the lower oxygen content in the catalysts and smaller metal particles and thus, the π–π stacking forces.^55,61^

Based on the UV–vis (high start and end absorbance values), ζ-potential (high negative value) and DLS studies (medium hydrodynamic radius, due to aggregation), a catalyst ink composition of isopropanol:water 5:5 (sample 6) was selected for the study of the different electrode deposition methods for both catalysts.

### 3.2 Physicochemical Electrode Characterization

**Figure 3:**
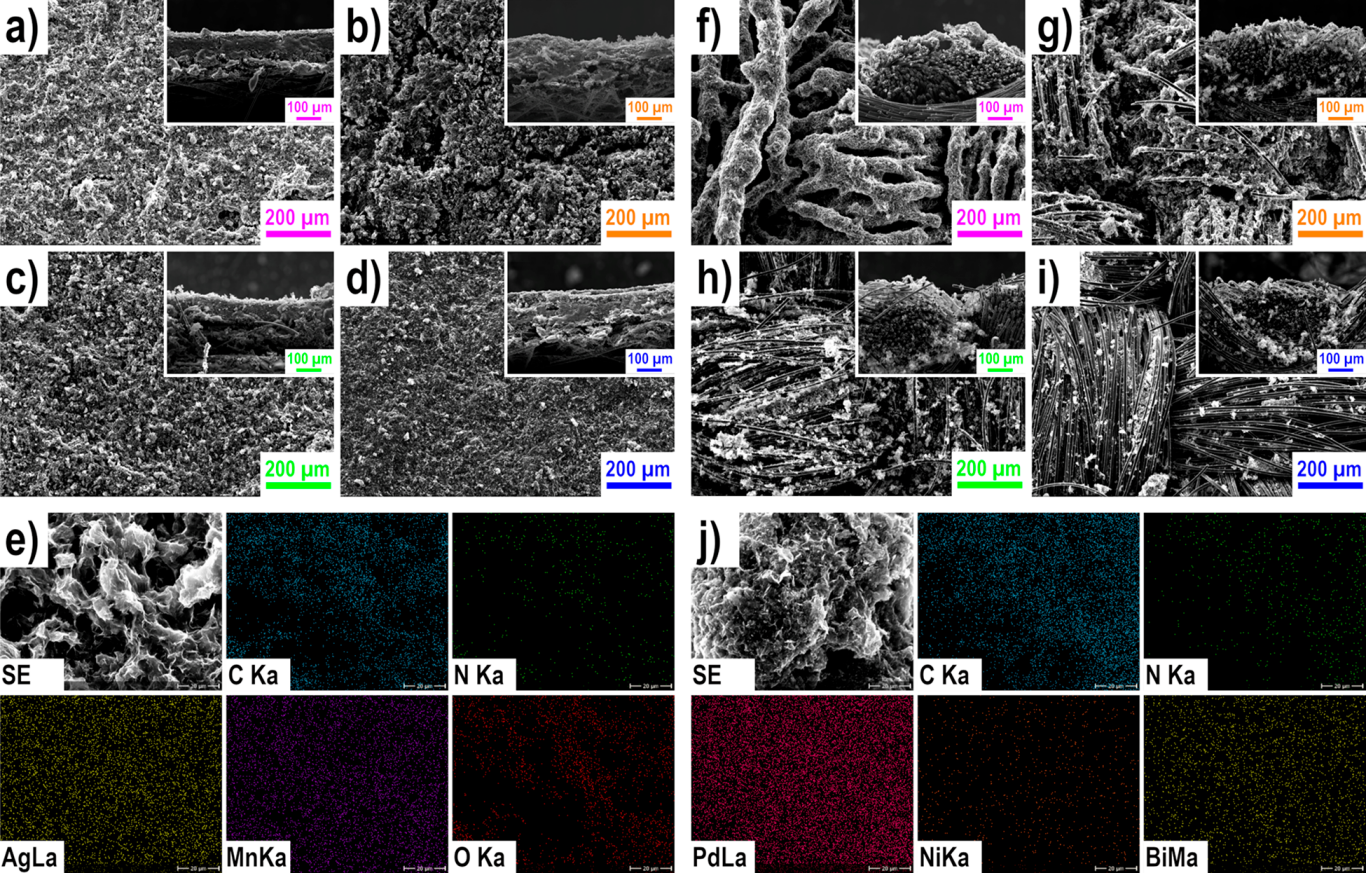
SEM images of the electrode coating (cross-section in the insets–scalebar = 100 μm): (a) spray_C_, (b) drop_C_, (c) brush_C_, (d) roll_C_, (f) spray_A_, (g) drop_A_, (h) brush_A_, and (i) roll_A_; and EDX-mapping of (e) brush_C_ and (j) spray_A_.

**Figure 4:**
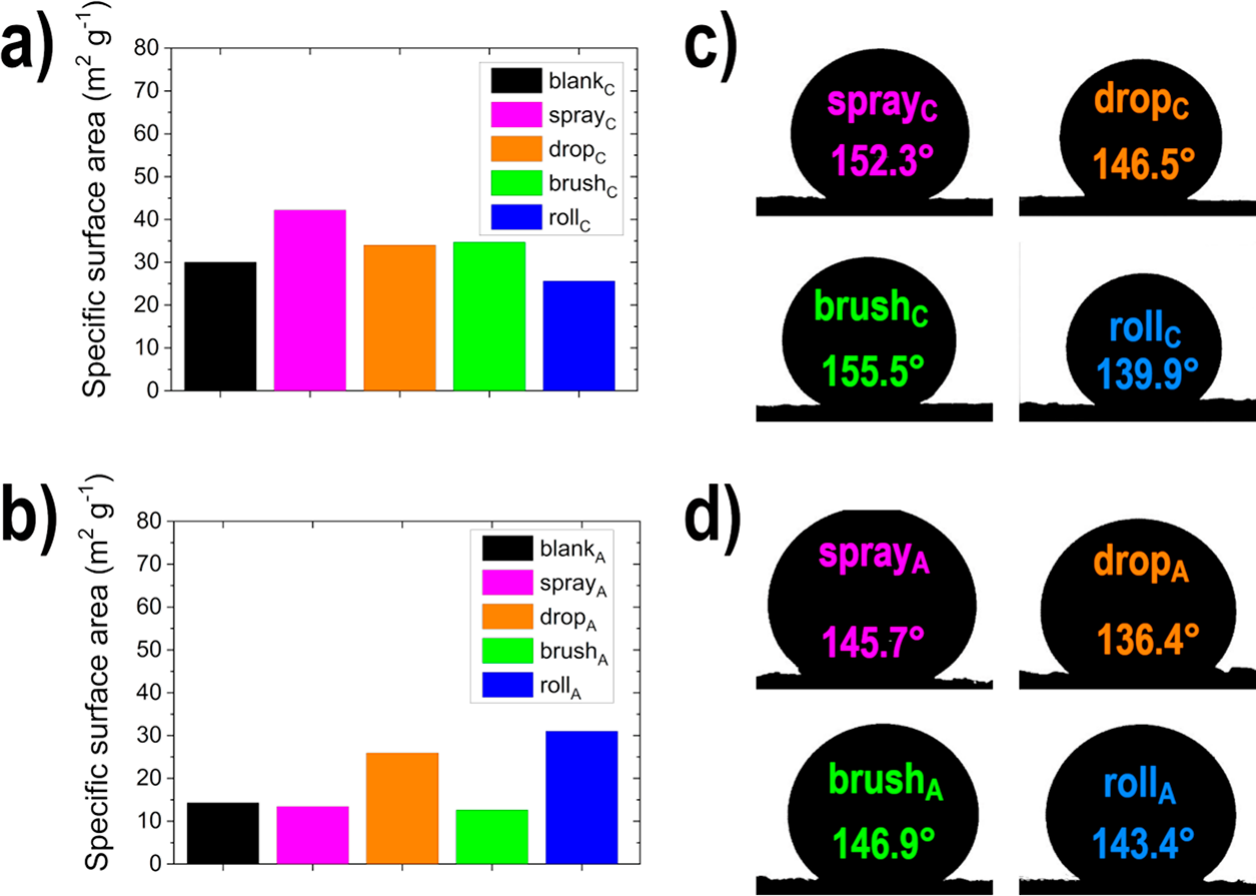
BET surface area results of (a) the cathodes and (b) the anodes; and water contact angles (θ) of the electrodes (c) cathodes, and (d) anodes.

The morphology of the electrode significantly influences the durability and the performance of fuel cells since the electrochemical processes occur at the catalyst layer–electrolyte interface. A high catalyst layer uniformity and distribution, together with a low aggregation quantity leads to reduced ohmic losses (and mass transport losses) and, thus, a higher power output.^64^ Therefore, the morphology of the catalyst layer, as well as the distribution of the active material on the N-rGO was analyzed with SEM coupled with EDX (see Figure 3, as well as Figures S1 and S2 and Table S3). In addition, the surface area was characterized with BET and the hydrophilicity with contact angle measurements (see Figure 4, as well as Table S4 in the Supporting Information).

The morphology of the prepared cathodic GDEs (Figures 3 a–d) on the carbon paper show clear differences in accordance to the electrode deposition method: the spray_C_ has a uniform dense morphology; the drop_C_ has large cracks but the catalyst layer is fluffier; the brush_C_ has a uniform porous catalyst layer structure; and the roll_C_ has a very densely packed catalyst layer. The cross-section images show that the N-rGO sheets are stacked vertically for spray_C_ and roll_C_, whereas for drop_C_ and brush_C_ a disordered randomly arrangement can be detected, which corresponds well to observations made from the surface.

In the SEM image (SE detector) in Figure 3e of the brush_C_, the single N-rGO sheets show a wrinkled 2D structure and are transparent (dark); if they stick together or form agglomerates, they appear white.^50^ Moreover, the large size (μm-range) of the N-rGO sheets, as determined with the hydrodynamic radius in the catalysts inks with DLS, can be seen. The influence of the electrode deposition method on the morphology, as described previously, becomes more visible at higher magnification levels (SE detector images in Figure 3e and Figure S1). The destruction of graphene-based materials during ultrasonication can be an issue as described in the literature.^7,65^ No destruction of the N-rGO structure through the ultrasonic nozzle during the spray-coating process was observed; nevertheless, no statement can be made about the dispersion of the catalyst inks in the ultrasonic bath since all have undergone the same procedure. The active material is independent of the electrode deposition method uniformly distributed over the entire surface area (Figure 3e and Figure S1) and the atomic ratio of the elements to each other is in a similar range for all prepared cathodic GDEs (see Table S3 in the Supporting Information).

The influence of the deposition method on the morphology of the electrode is even more evident in the SEM images of the anodes (Figures 3f–i), due to the use of a different GDL (carbon cloth vs carbon paper): the strands of the carbon cloth are evenly coated with catalyst only in spray_A_; brush_A_ shows coated and uncoated areas, as well as agglomerates; the agglomeration and noncoating is slightly more pronounced in drop_A_; and roll_A_ shows very poor coating on the surface and agglomerates under the surface. In the case of drop_A_, brush_A_, and roll_A_, large quantities of catalyst are pressed or flow through the carbon cloth mesh, as can be seen in the cross-section images. Conversely, this does not happen with spray_A_, where a fine catalyst layer can be seen on the surface of the threads. Jhong et al.^64^ also observed, when comparing different electrode deposition methods on carbon cloth for Pt/C catalyst cathodes for fuel cells, that agglomerates are formed when using a brush for deposition, this is due to agglomerate formation and growth inside the brush, which is then deposited. In the SE detector images in Figure 3j and Figure S2 in the Supporting Information of the anodic GDEs, a clear difference to the cathodes becomes apparent, i.e., the N-rGO sheets are much more restacked (most clearly at drop_A_ and roll_A_). The different active material, pure metal (anodic) in comparison to the transition-metal oxide hybrids (cathodic), results, as previously observed with the UV-vis and ζ potential measurements, in an increase in the π–π restacking forces of the N-rGO sheets.^61^ The EDX analyses resulted in a uniform distribution of active material and in comparable atomic ratios for all prepared anodic GDEs (see Figure 3j, Figure S2, and Table S3).

In addition to the visual investigation of the surface morphology, BET analyses (Figures 4a and 4b and Table S4) were used to describe the specific surface area (*S*_BET_) of the GDEs and the blank GDLs for comparison. The following trend for the *S*_BET_ values, which is in accordance with the observations from SEM, for the cathodic GDEs can be observed: spray_C_ (42.2 m^2^ g^–1^) > drop_C_ (34.0 m^2^ g^–1^) ≈ brush_C_ (34.7 m^2^ g^–1^) > roll_C_ (25.6 m^2^ g^–1^), whereas, the *S*_BET_ values are smaller for the anodic GDEs: spray_A_ (14.3 m^2^ g^–1^), drop_A_ (25.9 m^2^ g^–1^), brush_A_ (12.6 m^2^ g^–1^) and roll_A_ (31.0 m^2^ g^–1^). This can be attributed to the lower *S*_BET_ of the blank_A_ in comparison with blank_C_ and the higher restacking degree. For spray_A_ and brush_A_, no increase is observed after application, which is probably due to the coating of the pre-existing threads. Drop_A_ and roll_A_ show an enlargement, which is assumed to be the result of the large agglomerates between the threads, which were observed with SEM analysis. The BET analysis shows that, in every case (no matter whether cathode or anode, or which electrode deposition method was used), restacking has occurred, because, compared to the pure support material, a reduction was observed. Nosan et al.^57^ measured an *S*_BET_ value of 74 m^2^ g^–1^ for the same N-rGO material (not on a GDL).

The hydrophilicity requirements on the cathodic and anodic electrodes in the ADEFC are disparate. The catalyst layer at the cathode should be more hydrophobic, because of the gaseous oxygen, whereas the anode should be more hydrophilic because of the liquid ethanol and KOH mixture.^42,66^ The hydrophilicity of the GDE surfaces was thus investigated with contact angle measurements (see Figures 4c and 4d, as well as Table S4). The prepared cathodes become more hydrophilic in comparison to the blank GDL, whereas, when it came to the anodes, the hydrophilicity is reduced after deposition of the catalyst layer. Similar to the observations made previously in the catalyst ink measurements, this is caused by the different active material, more specifically on the oxygen content, on the N-rGO sheets.^55^ In addition, it can be observed that, regardless of active material (cathodic or anodic), the electrode deposition method and thus the emerging electrode morphology influences the hydrophilicity: the drop coating and roll coating production methods produce more hydrophilic electrode surfaces than ultrasonic spray coating or brush coating. The surface hydrophilicity or hydrophobicity is influenced by morphology (and also surface energy), which, in turn, depends on anchoring and surface tension.^67^

### 3.3 Electrochemical Electrode Characterization

The prepared electrodes were electrochemically characterized with the use of half-cell GDE measurements, to determine the oxidation and reduction processes (with cyclic voltammetry), as well as the ORR and EOR activity (with polarization curves). Half-cell GDE measurements are a helpful tool to investigate the electrodes at high current densities with a viable catalyst layer structure and three-phase boundary.^42,68^ Therefore, the influence of the electrode deposition method on the electrochemical behavior can be demonstrated. In addition to the half-cell GDE measurements, the best-performing electrodes were used for single-cell tests to determine their performance and durability.

**Figure 5:**
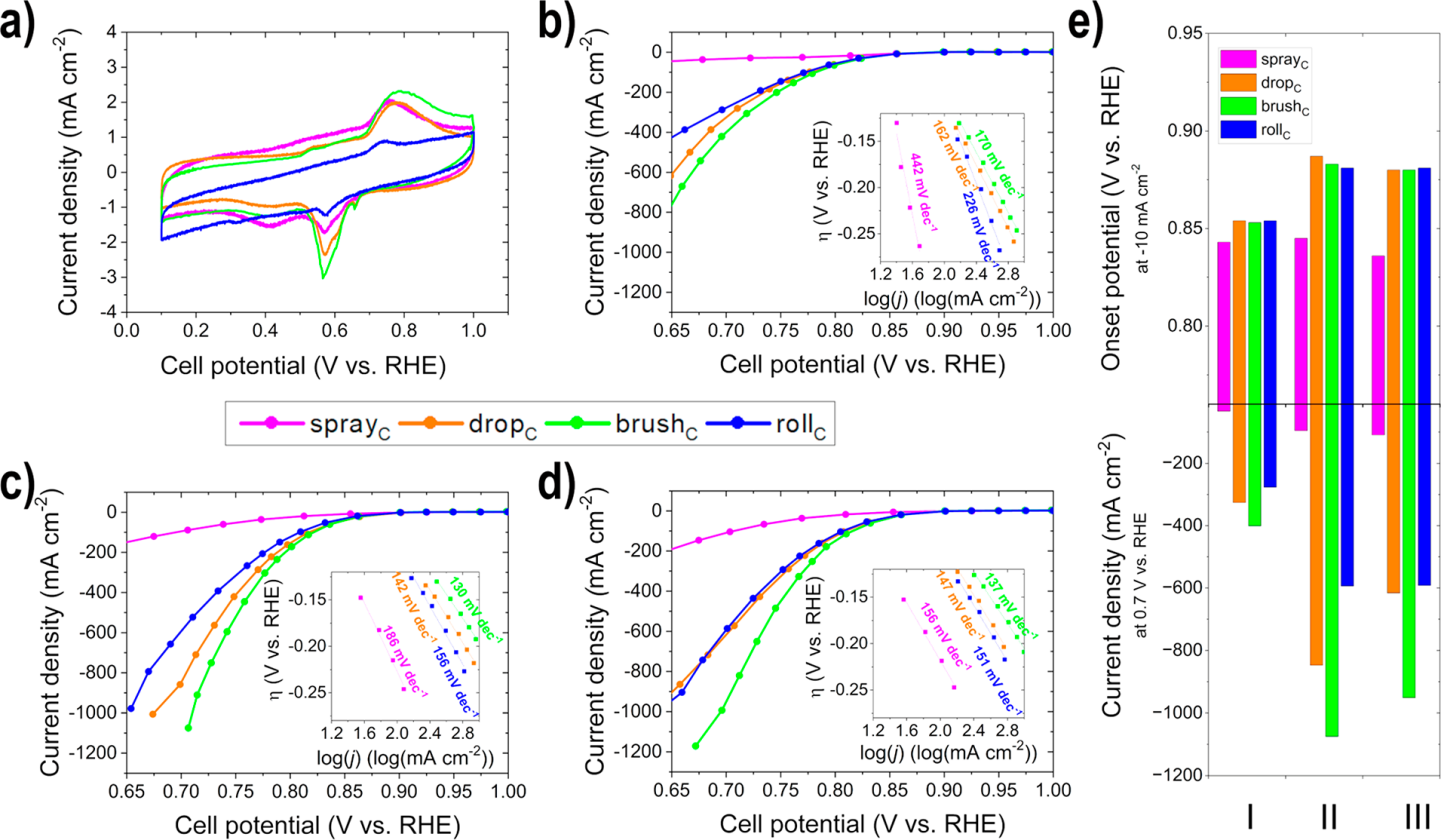
Half-cell GDE measurements of the produced cathodes (a) CVs; *iR*-compensated ORR polarization curves (Tafel plots in the insets: η = overpotential, *j* = current density) at (b) condition I, (c) condition II, and (d) condition III; and (e) comparison of the onset potentials (*E*_onset_) at −10 mA cm^–2^ and current density (*j*) at 0.7 V vs RHE for the different conditions.

In the cyclic voltammogramms (CVs) of the cathodic electrodes in Figure 5a, the oxidation and reduction peaks for manganese oxide (primarily visible: formation of MnOOH at 0.76 V vs RHE and 0.58 V vs RHE), but not for Ag, since they appear at a higher potential, can be seen.^29,69^ All of the produced cathodes show the same peaks; however, roll_C_ exhibits hysteresis in the baseline (high slope), due to charging currents and a higher resistance,^70^ which is indicated by the observed dense electrode and the restacking of the N-rGO sheets with SEM. The estimated double-layer capacitance (*C*_dl_) between 0.2–0.3 V vs RHE, where no faradaic processes occur, allows a statement about the surface area of the catalyst, since it is proportional to all conductive components.^42,71^ The trend of the *C*_dl_ values for the four different electrodes are consistent with the trend of the physical surface area measured with BET: spray_C_ (94 F g^–1^), drop_C_ (72 F g^–1^), brush_C_ (78 F g^–1^), and roll_C_ (59 F g^–1^). Thus, the influence of the electrode deposition method on the morphology (distribution and agglomeration) on the catalyst material could be additionally supported electrochemically.

The *iR*-compensated ORR polarization curves for the different conditions, shown in both Figure 5b–d and the values obtained therefrom in Figure 5e and Table S5, show a clear performance trend of the four prepared cathodic GDEs. The performance of all electrodes increases between condition I (25 °C) and condition II (60 °C) and stagnates between condition II and III (80 °C). The brush_C_ shows the highest current density (*j*) values at 0.7 V vs RHE in comparison to the other three electrodes. The onset potential (*E*_onset_) is in opposition for brush_C_, drop_C_, and roll_C_, and it is independent of the condition for all three in a similar range. Spray_C_, in contrast, shows significantly inferior values. The performance of the roll_C_ electrode improves more with condition (higher temperature) in comparison to that of brush_C_ or drop_C_, since reactant diffusion is increased with temperature, and the limitation caused by restacked N-rGO sheets (observed with SEM) is eliminated to some extent. The same can also be observed to a lesser extent for spray_C_. The Tafel analysis can be seen in the insets of Figure 5b–d and in Table S5 in the Supporting Information. The Tafel slope describes the kinetics of the observed reaction (how facile), whereas the product of *n* (number of electrons) and α (transfer coefficient) describes the efficiency of the catalytic interface.^72^ The Tafel slopes are the lowest and the *n*α values are the highest for the brush_C_ electrode, whereas the opposite applies to spray_C_. Therefore, the optimal catalyst layer utilization for the cathodic GDEs was achieved with the brush_C_ electrode. The low performance of the spray_C_ electrode is a result of the dense electrode layer structure. The observed restacking of N-rGO sheets with SEM and *S*_BET_ leads to a reduction of accessible catalytic active sites, surface area, and the diffusion pathways and thus leads to an efficiency loss.^9,45^ Grigoriev et al.^47^ observed that the graphene sheets irreversibly agglomerated and horizontally stacked during electrode production with a Pt/rGO catalyst for the polymer electrolyte membrane fuel cell (PEMFC), which resulted in lower activity.

**Figure 6:**
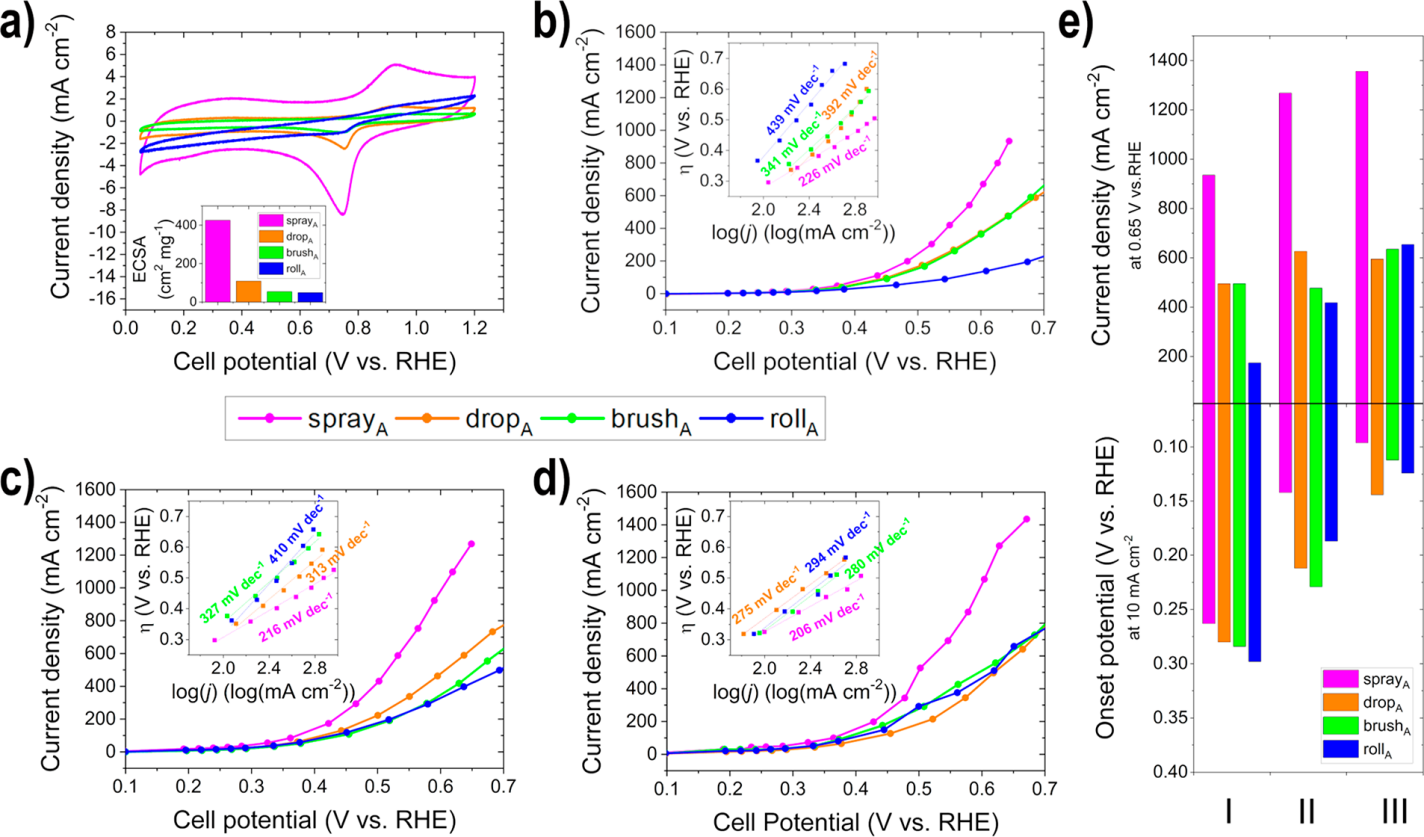
Half-cell GDE measurements of the produced anodes (a) CVs (ECSA in the inset); *iR*-compensated EOR polarization curves (Tafel plots in the insets: η = overpotential, *j* = current density) at (b) condition I, (c) condition II, and (d) condition III; and (e) comparison of the onset potentials (*E*_onset_) at 10 mA cm^–2^ and current density (*j*) at 0.65 V vs RHE for the different conditions.

The CVs of the anodic electrodes displayed in Figure 6a show the oxidation of Bi to Bi_2_O_3_ in the anodic scan at 0.9 V vs RHE and the reduction of PdO to Pd in the cathodic scan between 0.9–0.4 V vs RHE.^12,13,17,25^ The oxidation and reduction peaks of Ni (Ni(OH)_2_ ↔ NiOOH) are not visible in this potential range, as they occur between 1.2–1.5 V vs RHE;^25^ however, they can be found in Figure S3 in the Supporting Information. The PdO reduction peak was used to calculate the electrochemical active surface area (ECSA), as described in the literature.^13,15,20^ The electrodes were manufactured with the same metal loading; thus, any change in the ECSA can be attributed to the accessibility of the catalytic active material. Therefore, with spray_A_, the best active catalyst material utilization can be achieved, as it clearly shows the greatest ECSA (426 cm^2^ mg^–1^) compared to the other three electrodes or electrode deposition methods (drop_A_: 109 cm^2^ mg^–1^, brush_A_: 54 cm^2^ mg^–1^ and roll_A_: 49 cm^2^ mg^–1^). The high ECSA is due to the good accessibility of the active sites, as well as the low agglomeration of the catalyst material,^64^ as observed by the SEM images. The roll_A_ CV shows similarly as roll_C_ a modification of the flat baseline, and thus this phenomenon can be attributed to the electrode deposition method. The estimated *C*_dl_ at 0.44 V vs RHE follows the same trend as the ECSA: spray_A_ (135 F g^–1^), drop_A_ (36 F g^–1^), brush_A_ (20 F g^–1^) and roll_A_ (28 F g^–1^). The *C*_dl_ for the anodes, in contrast to the cathodes is not consistent with the *S*_BET_ values, which can be attributed to the different surface structure of the substrate used and the observed restacking and agglomeration. This results in nonutilizable active material (enclosed between sheets) and thus differences in the various values, that describe the different surface areas (as described before) of ECSA, *C*_dl_, and *S*_BET_.

The activity of the prepared anodic GDEs increases for nearly all the electrode deposition methods with increasing temperature (condition I to III) due to improved EOR kinetics^42,66^ and results in higher *j*, lower *E*_onset_, lower OCP, higher *n*α values, and lower Tafel slopes, as shown in Figure 6b–e and Table S5 in the Supporting Information. In each of the tested EOR conditions, spray_A_ definitely shows the best performance and activity and thus the highest catalyst layer utilization. The lower performance of drop_A_, brush_A_, and roll_A_, when compared to spray_A_, depends on the lower ECSA values of the electrodes, caused by the observed agglomeration, restacking and the inaccessible active catalyst material,^64^ as described before. The performance of the roll_A_ electrode improves significantly (as previously observed with roll_C_) with higher temperature, due to increased reactant diffusion. The performance of drop_A_ and brush_A_ is quite comparable for all tested conditions, due to the similar morphology observed with SEM.

Remarkably, the influence of the electrode deposition method has quite the opposite effect in terms of activity for the cathode and anode, e.g., the anode prepared with ultrasonic spray coating achieved the highest performance. However, the cathode following the same method was clearly the worst performing. This is due to the use of different GDLs with unequal substrate surface structures and the different aggregate state of the reactant and thus the hydrophilic requirements, as previously mentioned.

The electrodes for the MEA used for the single cell tests were thus brush_C_ and spray_A_. The maximum power density (*p*_max_) values for the different conditions can be found in Table 1.

**Table 1:** Maximum Power Density Results of the Single Cell Tests for Conditions I–III

condition	N-rGO based MEA	comm. MEA
I	21.4 mW cm^–2^	1.88 mW cm^–2^
II	49.0 mW cm^–2^	20.9 mW cm^–2^
III	62.6 mW cm^–2^	22.8 mW cm^–2^

**Figure 7:**
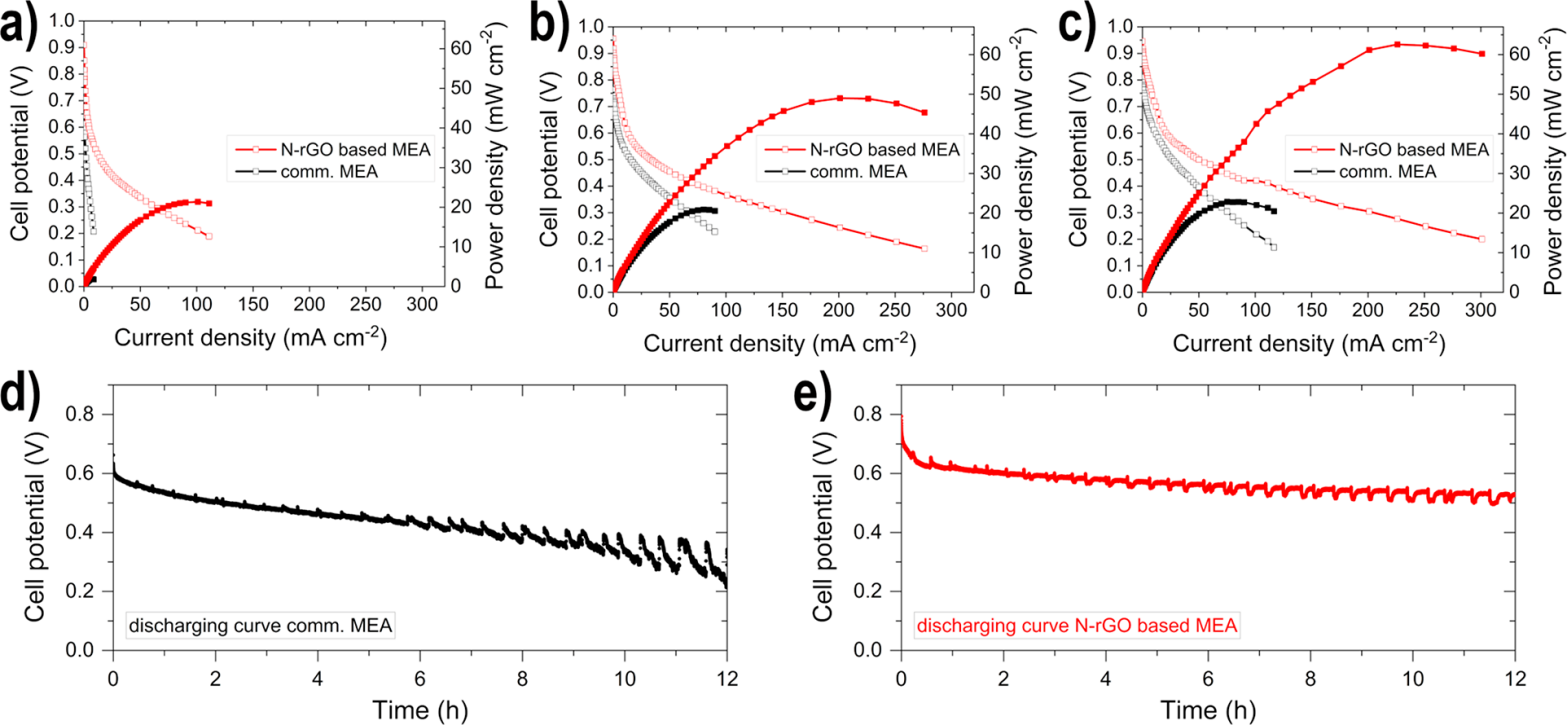
Polarization (unfilled symbols) and power density curves (filled symbols) of the ADEFC tests at (a) condition I, (b) condition II, and (c) condition III. The discharging curves of the durability study for (d) the comm. MEA and (e) the N-rGO-based MEA.

The *p*_max_ values increase from condition I to III, due to increasing temperature and therefore, the membrane conductivity, the electrode kinetics, and the mass transfer properties are enhanced.^42,66^ The N-rGO MEA is superior to the comm. MEA under any operating condition. The higher performance of the N-rGO based MEA, in comparison to the comm. MEA (Figure 7a–c), can be attributed to several factors: (i) the inclusion of N-rGONRs in the CS-membrane due to the resulting hydrophilic regions (oxygen groups), which enable easier hydroxide transport and reduced ethanol crossover due to hydrophobic domains (*sp*^2^ carbon atoms),^37,38^ (ii) the ethanol tolerance of the cathode catalyst due to the utilization of Ag–Mn_*x*_O_*y*_ in contrast to Pt,^29^ (iii) the adatoms (Ni and Bi) in the Pd-based anodic catalyst, due to favored OH^–^ adsorption on the surface,^25,56^ and (iv) the utilization of N-rGO as catalyst support, due to better distribution of the active material and the improved oxidative removal of EOR intermediates.^8,22^ Moreover, the improvement in ethanol crossover suppression and the ethanol tolerance of the cathode is also reflected in the much higher OCV of the N-rGO MEA compared to the comm. MEA (e.g., condition I: 0.909 V vs 0.705 V, respectively). The OCV is lower for the comm. MEA, as the Pt/C catalyst shows EOR activity, and thus in the case of ethanol crossover, leads to mixed potentials.^29,39,42^

The durability study of the N-rGO based MEA (Figure 7e) resulted in a smaller (half as much) degradation rate (*d*) of 0.3 mW cm^–2^ h^–1^ for the 12 h measurement period in comparison to the comm. MEA (Figure 7d), which was 0.6 mW cm^–2^ h^–1^. A higher ethanol crossover rate through the membrane leads to unused fuel for oxidation and thus activity losses, which is reduced through the incorporation of N-rGONRs in the membrane, as described previously.^37,38^ The ethanol crossover from the anode to the cathode results moreover, in a larger potential loss for Pt-catalysts, in contrast to the Ag–Mn_*x*_O_*y*_/N-rGO catalyst.^29^ Therefore, we expect that the visible voltage fluctuations, which are clearly increasing for the comm. MEA are due to the occurrence of an ethanol crossover and to the accumulation of ethanol oxidation intermediates on the anodic catalyst surface. This is because, in contrast to the N-rGO MEA, there is no N-rGO, Ni, and Bi present, which supports the oxidation removal of intermediates.^8,21,22,25,56^ In addition, the morphology of the electrodes was analyzed after the durability study with SEM and EDX, as shown in the Supporting Information (Table S6, Figure S4 and S5). Compared to the freshly manufactured electrodes, there is nearly no difference in the morphology and the atomic ratio of the elements to each other, as well as the even distribution of the active material is again observed for both GDEs. Hence, the degradation in the last 6 h of the measurement is remarkably reduced for the N-rGO based MEA and leads to a small *d* of 0.03 mW cm^–2^ h^–1^, in contrast to the comm. MEA. Hou et al.^60^ observed a *d* of 0.027 mW cm^–2^ h^–1^ during a durability study of 336 h. Thus, a very high long-term durability of the N-rGO based MEA can be assumed.

The comm. MEA was prepared as a reference for comparison, as the literature data of graphene compounds in the ADEFC is rare and complex: the conditions are not reported as sufficient or are different from the conditions used in this work. However, a literature performance comparison of various graphene compounds containing MEA components can be found in Table 2. The competitiveness of the produced N-rGO based MEA is highlighted considering the use of completely Pt-free catalysts with low loadings and the biobased membrane material with a high *p*_max_ of 62.6 mW cm^–2^ at 80 °C.

**Table 2:** Comparison of the ADEFC Performances of Various Graphene-Species Containing MEA Components (in Bold)^a^

anode catalyst (loading)	cathode catalyst (loading)	membrane	anode fuel	*T* (°C)	*p*_max_ (mW cm^–2^)	ref
**PdNi/EGO (1 mg**_**Pd**_**cm**^**–2**^**)**	Pt/C	PTFE re-enforced Toku-yama AHA membrane	1 M EtOH	50	16.6	(13)
			1 M NaOH			
Pd/C (2.56 mg_cat_ cm^–2^)	**(Bg-CA-M)-Fe/N/C@800 °C (2.56mg cm**^**–2**^**)**	Tokuyama A-201	2 M EtOH	90	64	(27)
			1 M KOH			
**Ni@Au@Pd/rGO (0.5 mg**_**Pd**_**cm**^**–2**^**)**	Pt/C (1 mg_Pt_ cm^–2^)	Tokuyama A901	N/A	80	185.78	(18)
**Pd**_**1**_**Pt**_**0.98**_**/GA/NF**	Pt/C	N/A	4 M EtOH	RT	3.6	(19)
			5 M KOH			
Pt–Ru catalyst (4 mg cm^–2^)	Pt catalyst (4 mg cm^–2^)	**QPVA/GO**	2 M EtOH	60	11.4	(36)
			2 M KOH			
Pt–Ru/C (2 mg_metal_ cm^–2^)	Pt/C (1 mg_metal_ cm^–2^)	**CS + Mg + GO**	3 M EtOH	80	57.6 ± 3.7	(37)
			5 M KOH			
PdNiBi/C (0.75 mg_metal_ cm^–2^)	PtRu/C (0.5 mg_metal_ cm^–2^)	**CS/N-rGONRs**	3 M EtOH	57	34.5	(38)
			5 M KOH			
**Pd/NRGO (1 mg**_**metal**_**cm**^**–2**^**)**	**Pd/NRGO (1 mg**_**metal**_**cm**^**–2**^**)**	Tokuyama A-006	4 M EtOH	RT	31.5	(28)
			2 M KOH			
**PtAl/rGO**	N/A	N/A	2 M EtOH	50	27.07	(23)
			1 M NaOH			
**Pd/G-US (1 mg**_**Pd**_**cm**^**–2**^**)**	Pt/C (1 mg_Pt_ cm^–2^)	Nafion 117	2 M EtOH	80	38	(24)
			1 M KOH			
Pt/C (10 mg cm^–2^)	**Fe–N–C-3h (2mg cm**^**–2**^**)**	Tokuyama A201	2 M EtOH	RT	23.97	(32)
			2 M KOH			
PtRu/C (1 mg cm^–2^)	**Co/SN-rGO (1.26mg cm**^**–2**^**)**	commercial PBI Danish Power Systems	2 M EtOH	90	100	(30)
			1 M KOH			
**PdNiBi/N-rGO (0.5 mg**_**active m.**_**cm**^**–2**^**)**	**Ag–Mn**_***x***_**O**_**y**_**/N-rGO (0.25 mg**_**active m.**_**cm**^**–2**^**)**	**CS/N-rGONRs**	3 M EtOH	80	62.6	this work
			5 M KOH			

a Legend: *T*, temperature; *p*_max_, maximum power density.

## 4 Conclusion

The influence of the electrode deposition method (ultrasonic spray coating, drop coating, brush coating, and roll coating) of N-rGO based catalyst inks—Ag–Mn_*x*_O_*y*_/N-rGO for the cathode and PdNiBi/N-rGO for the anode—on the morphology and thus on the performance in the ADEFC was successfully determined, whereby restacking of graphene sheets has a major effect. Therefore, the dispersion behavior and the stability of the catalyst inks was evaluated first in various solvent mixtures. It was shown that the nanoparticles on the N-rGO sheets affects the π–π restacking forces and thus the stability of the ink. The most optimal ratio of isopropanol to water for a stable and well dispersed ink is 1:1. The properties of the two different catalysts (anode or cathode), such as a tendency toward restacking or agglomeration, were also observed in the produced GDEs, which influenced the electrochemical activity: the brush coated cathode and the ultrasonic spray coated anode achieved the highest levels of performance. The different GDLs and the different requirements for hydrophobicity and hydrophilicity for the anode and cathode affect both the structure and morphology of the catalyst layer and which method of electrode deposition is most suitable. The completely N-rGO based MEA, which is Pt-free and includes a biobased membrane, produced with the best-performing cathode and anode reached maximum power densities of 62.6 mW cm^–2^ at 80 °C—almost triple the power output in comparison with the commercial AEM and catalysts. Moreover, the incorporation of graphene-compounds into the MEA resulted in good long-term durability, with a small degradation rate of 0.03 mW cm^–2^ h^–1^ compared to the commercial MEA (double). Therefore, the influence of the active material on the N-rGO sheets on the restacking and the impact of the surface texture of the GDL during electrode deposition were demonstrated, paving the way for further improvement in the use of graphene-based materials for fuel cell electrode production.

## Supporting Information Available

Physical investigation results of the PdNiBi/N-rGO catalyst inks (Table S1); Physical investigation results of the Ag–Mn_*x*_O_*y*_/N-rGO catalyst inks (Table S2); EDX results (in wt %) of all produced electrodes (Table S3); EDX mapping of the produced cathodes ((a) spray_C_, (b) drop_C_, and (c) roll_C_) (Figure S1); EDX mapping of the produced anodes ((a) drop_A_, b) brush_A_ and c) roll_A_ (Figure S2); Brunauer–Emmett–Teller results (*S*_*BET*_: BET surface area) and contact angles (θ) of all cathodic and anodic electrodes in comparison with the blank gas diffusion layers (Table S4); Cyclic voltammogramms of the PdNiBi/N-rGO electrodes with a scan rate of 10 mV s^–1^ in a potential range from 0.05 V – 1.50 V vs RHE in 5 M KOH at RT (Figure S3); Electrochemical half-cell GDE measurement results and Tafel plot values for all cathodic and anodic electrodes (Table S5); EDX results (in wt %) of the electrodes of the N-rGO based MEA after the durability study (Table S6); (a) SEM images of the electrode coating and (b) EDX mapping of the cathodic electrode of the N-rGO based MEA after the durability study (Figure S4); (a) SEM images of the electrode coating and (b) EDX mapping of the anodic electrode of the N-rGO based MEA after the durability study (Figure S5)PDF)

## References

[ref1] Iwan A.; Malinowski M.; Pasciak G. Polymer Fuel Cell Components Modified by Graphene: Electrodes, Electrolytes and Bipolar Plates. Renew. Sustain. Energy Rev. 2015, 49, 954–967. 10.1016/j.rser.2015.04.093.

[ref2] Ji L.; Meduri P.; Agubra V.; Xiao X.; Alcoutlabi M. Graphene-Based Nanocomposites for Energy Storage. Adv. Energy Mater. 2016, 6 (16), 1502159 10.1002/aenm.201502159.

[ref3] Tsang A. C. H.; Kwok H. Y. H.; Leung D. Y. C. The Use of Graphene Based Materials for Fuel Cell, Photovoltaics, and Supercapacitor Electrode Materials. Solid State Sci. 2017, 67, A1–A14. 10.1016/j.solidstatesciences.2017.03.015.

[ref4] Farooqui U. R.; Ahmad A. L.; Hamid N. A. Graphene Oxide: A Promising Membrane Material for Fuel Cells. Renew. Sustain. Energy Rev. 2018, 82, 714–733. 10.1016/j.rser.2017.09.081.

[ref5] Iqbal M. Z.; Rehman A. U.; Siddique S. Prospects and Challenges of Graphene Based Fuel Cells. J. Energy Chem. 2019, 39, 217–234. 10.1016/j.jechem.2019.02.009.

[ref6] Hossain S.; Abdalla A. M.; Suhaili S. B. H.; Kamal I.; Shaikh S. P. S.; Dawood M. K.; Azad A. K. Nanostructured Graphene Materials Utilization in Fuel Cells and Batteries: A Review. J. Energy Storage 2020, 29, 101386 10.1016/j.est.2020.101386.

[ref7] Tsang C. H. A.; Huang H.; Xuan J.; Wang H.; Leung D. Y. C. Graphene Materials in Green Energy Applications: Recent Development and Future Perspective. Renew. Sustain. Energy Rev. 2020, 120, 109656 10.1016/j.rser.2019.109656.

[ref8] Su H.; Hu Y. H. Recent Advances in Graphene-Based Materials for Fuel Cell Applications. Energy Sci. Eng. 2021, 9 (7), 958–983. 10.1002/ese3.833.

[ref9] Yadav R.; Subhash A.; Chemmenchery N.; Kandasubramanian B. Graphene and Graphene Oxide for Fuel Cell Technology. Ind. Eng. Chem. Res. 2018, 57 (29), 9333–9350. 10.1021/acs.iecr.8b02326.

[ref10] Ren W. Preparation of Graphene Electrode. In Graphene for Flexible Lighting and Displays; Woodhead Publishing, 2020; Vol. 15, pp 27–57. 10.1016/B978-0-08-102482-9.00003-4.

[ref11] Sarapuu A.; Kibena-Põldsepp E.; Borghei M.; Tammeveski K. Electrocatalysis of Oxygen Reduction on Heteroatom-Doped Nanocarbons and Transition Metal-Nitrogen-Carbon Catalysts for Alkaline Membrane Fuel Cells. J. Mater. Chem. A 2018, 6 (3), 776–804. 10.1039/C7TA08690C.

[ref12] Krishna R.; Fernandes D. M.; Ventura J.; Freire C.; Titus E. Facile Synthesis of Reduced Graphene Oxide Supported Pd@NixB/RGO Nanocomposite: Novel Electrocatalyst for Ethanol Oxidation in Alkaline Media. Int. J. Hydrogen Energy 2016, 41 (27), 11811–11822. 10.1016/j.ijhydene.2015.12.034.

[ref13] Tan J. L.; De Jesus A. M.; Chua S. L.; Sanetuntikul J.; Shanmugam S.; Tongol B. J. V.; Kim H. Preparation and Characterization of Palladium-Nickel on Graphene Oxide Support as Anode Catalyst for Alkaline Direct Ethanol Fuel Cell. Appl. Catal. A Gen. 2017, 531, 29–35. 10.1016/j.apcata.2016.11.034.

[ref14] Bhunia K.; Khilari S.; Pradhan D. Monodispersed PtPdNi Trimetallic Nanoparticles-Integrated Reduced Graphene Oxide Hybrid Platform for Direct Alcohol Fuel Cell. ACS Sustain. Chem. Eng. 2018, 6 (6), 7769–7778. 10.1021/acssuschemeng.8b00721.

[ref15] Yazdan-Abad M. Z.; Noroozifar M.; Alfi N.; Modarresi-Alam A. R.; Saravani H. Pd Nanonetwork Decorated on RGO as a High-Performance Electrocatalyst for Ethanol Oxidation. Appl. Surf. Sci. 2018, 462 (July), 112–117. 10.1016/j.apsusc.2018.07.201.

[ref16] Rajesh D.; Indra Neel P.; Pandurangan A.; Mahendiran C. Pd-NiO Decorated Multiwalled Carbon Nanotubes Supported on Reduced Graphene Oxide as an Efficient Electrocatalyst for Ethanol Oxidation in Alkaline Medium. Appl. Surf. Sci. 2018, 442, 787–796. 10.1016/j.apsusc.2018.02.174.

[ref17] Rezaee S.; Shahrokhian S.; Amini M. K. Nanocomposite with Promoted Electrocatalytic Behavior Based on Bimetallic Pd-Ni Nanoparticles, Manganese Dioxide, and Reduced Graphene Oxide for Efficient Electrooxidation of Ethanol. J. Phys. Chem. C 2018, 122 (18), 9783–9794. 10.1021/acs.jpcc.8b01475.

[ref18] Wang F.; Qiao J.; Wang J.; Wu H.; Yue X.; Wang Z.; Sun W.; Sun K. Reduced Graphene Oxide Supported Ni@Au@Pd Core@bishell Nanoparticles as Highly Active Electrocatalysts for Ethanol Oxidation Reactions and Alkaline Direct Bioethanol Fuel Cells Applications. Electrochim. Acta 2018, 271, 1–9. 10.1016/j.electacta.2018.03.013.

[ref19] Tsang C. H. A.; Leung D. Y. C. Use of Pd-Pt Loaded Graphene Aerogel on Nickel Foam in Direct Ethanol Fuel Cell. Solid State Sci. 2018, 75, 21–26. 10.1016/j.solidstatesciences.2017.11.005.

[ref20] Yao C.; Zhang Q.; Su Y.; Xu L.; Wang H.; Liu J.; Hou S. Palladium Nanoparticles Encapsulated into Hollow N-Doped Graphene Microspheres as Electrocatalyst for Ethanol Oxidation Reaction. ACS Appl. Nano Mater. 2019, 2 (4), 1898–1908. 10.1021/acsanm.8b02294.

[ref21] Zhang J.; Zhao Z.; Wang Y.; Wang J.; Peng D.; Li B.; Bo T.; Zheng K.; Zhou Z.; Lv L.; Xin Z.; Zhang B.; Shao L. Ethanol Electrooxidation on Highly Active Palladium/Graphene Oxide Aerogel Catalysts. Chem. Phys. 2020, 534, 110753 10.1016/j.chemphys.2020.110753.

[ref22] Chowdhury S. R.; Maiyalagan T.; Bhattachraya S. K.; Gayen A. Influence of Phosphorus on the Electrocatalytic Activity of Palladium Nickel Nanoalloy Supported on N-Doped Reduced Graphene Oxide for Ethanol Oxidation Reaction. Electrochim. Acta 2020, 342, 136028 10.1016/j.electacta.2020.136028.

[ref23] Vu T. H. T.; Nguyen M. D.; Mai A. T. N. Influence of Solvents on the Electroactivity of PtAl/RGO Catalyst Inks and Anode in Direct Ethanol Fuel Cell. J. Chem. 2021, 2021, 1–15. 10.1155/2021/6649089.

[ref24] Souza F. M.; Lima T. S.; Böhnstedt P.; Pinheiro V. S.; Batista B. L.; Parreira L. S.; Simões F. R.; Codognoto L.; Hammer P.; Santos M. C. Fast and Inexpensive Synthesis of Multilayer Graphene Used as Pd Support in Alkaline Direct Ethanol Fuel Cell Anode. Electrocatalysis 2021, 12 (6), 715–730. 10.1007/s12678-021-00685-4.

[ref25] Wolf S.; Roschger M.; Genorio B.; Hodnik N.; Gatalo M.; Ruiz-zepeda F.; Hacker V. Reduced Graphene Oxide as Efficient Carbon Support for Pd-Based Ethanol Oxidation Catalysts in Alkaline Media. J. Electrochem. Sci. Eng. 2023, 13, 771-782.. 10.5599/jese.1643.

[ref26] Lee K.; Ahmed M. S.; Jeon S. Electrochemical Deposition of Silver on Manganese Dioxide Coated Reduced Graphene Oxide for Enhanced Oxygen Reduction Reaction. J. Power Sources 2015, 288, 261–269. 10.1016/j.jpowsour.2015.04.060.

[ref27] Rauf M.; Chen R.; Wang Q.; Wang Y. C.; Zhou Z. Y. Nitrogen-Doped Carbon Nanotubes with Encapsulated Fe Nanoparticles as Efficient Oxygen Reduction Catalyst for Alkaline Membrane Direct Ethanol Fuel Cells. Carbon 2017, 125, 605–613. 10.1016/j.carbon.2017.09.093.

[ref28] Kakaei K.; Rahnavardi M. Synthesis of Nitrogen-Doped Reduced Graphene Oxide and Its Decoration with High Efficiency Palladium Nanoparticles for Direct Ethanol Fuel Cell. Renew. Energy 2021, 163, 1277–1286. 10.1016/j.renene.2020.09.043.

[ref29] Wolf S.; Roschger M.; Genorio B.; Kolar M.; Garstenauer D.; Bitschnau B.; Hacker V. Ag-MnxOy on Graphene Oxide Derivatives as Oxygen Reduction Reaction Catalyst in Alkaline Direct Ethanol Fuel Cells. Catalysts 2022, 12 (7), 780 10.3390/catal12070780.

[ref30] Fajardo S.; Ocón P.; Rodríguez J. L.; Pastor E. Co Supported on N and S Dual-Doped Reduced Graphene Oxide as Highly Active Oxygen-Reduction Catalyst for Direct Ethanol Fuel Cells. Chem. Eng. J. 2023, 461, 142053 10.1016/j.cej.2023.142053.

[ref31] Wolf S.; Roschger M.; Genorio B.; Garstenauer D.; Hacker V. Mixed Transition-Metal Oxides on Reduced Graphene Oxide as a Selective Catalyst for Alkaline Oxygen Reduction. ACS Omega 2023, 8 (12), 11536–11543. 10.1021/acsomega.3c00615. 37008156 PMC10061501

[ref32] Jafari M.; Gharibi H.; Kazemi M.; Heydari A.; Zhiani M.; Parnian M. J. Metal-Nitrogen Co-Doped Hierarchical Porous Carbon Derived from the Bimetallic Metal-Organic Framework as ORR Electrocatalyst for Passive Alkaline Direct Ethanol Fuel Cell. J. Electroanal. Chem. 2022, 920, 116620 10.1016/j.jelechem.2022.116620.

[ref33] Seyed Bagheri S. M.; Gharibi H.; Zhiani M. Introduction of a New Active and Stable Cathode Catalyst Based on Bimetal-Organic Frameworks/PPy-Sheet for Alkaline Direct Ethanol Fuel Cell. Int. J. Hydrogen Energy 2022, 47 (56), 23552–23569. 10.1016/j.ijhydene.2022.05.142.

[ref34] Mashkani F. A.; Gharibi H.; Amani M.; Zhiani M.; Morsali A. A Novel Electrocatalyst Based on Fe-ZIF-PPY Nanocomposite for Oxygen Reduction Reaction in Air-Breathing Direct-Ethanol Fuel Cell. Appl. Surf. Sci. 2022, 584, 152529 10.1016/j.apsusc.2022.152529.

[ref35] Zakaria Z.; Shaari N.; Kamarudin S. K. Preliminary Study of Alkaline Direct Ethanol Fuel Cell by Using Crosslinked Quaternized Poly (Vinyl Alcohol)/Graphene Oxide Membrane. J. Kejuruter. 2018, 30 (2), 219–227. 10.17576/jkukm-2018-30(2)-12.

[ref36] Zakaria Z.; Kamarudin S. K.; Timmiati S. N. Influence of Graphene Oxide on the Ethanol Permeability and Ionic Conductivity of QPVA-Based Membrane in Passive Alkaline Direct Ethanol Fuel Cells. Nanoscale Res. Lett. 2019, 14 (28), 1–18. 10.1186/s11671-018-2836-3. 30659414 PMC6338673

[ref37] Kaker B.; Hribernik S.; Mohan T.; Kargl R.; Stana Kleinschek K.; Pavlica E.; Kreta A.; Bratina G.; Lue S. J.; Božič M. Novel Chitosan-Mg(OH)2-Based Nanocomposite Membranes for Direct Alkaline Ethanol Fuel Cells. ACS Sustain. Chem. Eng. 2019, 7 (24), 19356–19368. 10.1021/acssuschemeng.9b02888.

[ref38] Gorgieva S.; Osmić A.; Hribernik S.; Božič M.; Svete J.; Hacker V.; Wolf S.; Genorio B. Efficient Chitosan/Nitrogen-Doped Reduced Graphene Oxide Composite Membranes for Direct Alkaline Ethanol Fuel Cells. Int. J. Mol. Sci. 2021, 22 (4), 1740 10.3390/ijms22041740. 33572312 PMC7916145

[ref39] Zhao T. S.; Li Y. S.; Shen S. Y. Anion-Exchange Membrane Direct Ethanol Fuel Cells: Status and Perspective. Front. Energy Power Eng. China 2010, 4 (4), 443–458. 10.1007/s11708-010-0127-5.

[ref40] Yu E. H.; Wang X.; Krewer U.; Li L.; Scott K. Direct Oxidation Alkaline Fuel Cells: From Materials to Systems. Energy Environ. Sci. 2012, 5 (2), 5668–5680. 10.1039/C2EE02552C.

[ref41] Monyoncho E. A.; Woo T. K.; Baranova E. A. Ethanol Electrooxidation Reaction in Alkaline Media for Direct Ethanol Fuel Cells. SPR Electrochemistry 2018, 15, 1–57. 10.1039/9781788013895-00001.

[ref42] Roschger M.; Wolf S.; Mayer K.; Billiani A.; Genorio B.; Gorgieva S.; Hacker V. Influence of the Electrocatalyst Layer Thickness on Alkaline DEFC Performance. Sustain. Energy Fuels 2023, 7 (4), 1093–1106. 10.1039/D2SE01729F. 36818600 PMC9926948

[ref43] Strong A.; Thornberry C.; Beattie S.; Chen R.; Coles S. R. Depositing Catalyst Layers in Polymer Electrolyte Membrane Fuel Cells: A Review. J. Fuel Cell Sci. Technol. 2015, 12 (6), 064001 10.1115/1.4031961.

[ref44] Marinkas A.; Arena F.; Mitzel J.; Prinz G. M.; Heinzel A.; Peinecke V.; Natter H. Graphene as Catalyst Support: The Influences of Carbon Additives and Catalyst Preparation Methods on the Performance of PEM Fuel Cells. Carbon 2013, 58, 139–150. 10.1016/j.carbon.2013.02.043.

[ref45] Liu J.; Takeshi D.; Sasaki K.; Lyth S. M. Defective Graphene Foam: A Platinum Catalyst Support for PEMFCs. J. Electrochem. Soc. 2014, 161 (9), F838–F844. 10.1149/2.0231409jes.

[ref46] Shui J.; Wang M.; Du F.; Dai L. N-Doped Carbon Nanomaterials Are Durable Catalysts for Oxygen Reduction Reaction in Acidic Fuel Cells. Sci. Adv. 2015, 1 (1), e1400129 10.1126/sciadv.1400129. 26601132 PMC4644083

[ref47] Grigoriev S. A.; Fateev V. N.; Pushkarev A. S.; Pushkareva I. V.; Ivanova N. A.; Kalinichenko V. N.; Presnyakov M. Y.; Wei X. Reduced Graphene Oxide and Its Modifications as Catalyst Supports and Catalyst Layer Modifiers for PEMFC. Materials 2018, 11 (8), 1405 10.3390/ma11081405. 30103437 PMC6119945

[ref48] Galvan V.; Glass D. E.; Baxter A. F.; Surya Prakash G. K. Reduced Graphene Oxide Supported Palladium Nanoparticles for Enhanced Electrocatalytic Activity toward Formate Electrooxidation in an Alkaline Medium. ACS Appl. Energy Mater. 2019, 2 (10), 7104–7111. 10.1021/acsaem.9b01020.

[ref49] Park J. C.; Park S. H.; Chung M. W.; Choi C. H.; Kho B. K.; Woo S. I. Optimization of Catalyst Layer Composition for PEMFC Using Graphene-Based Oxygen Reduction Reaction Catalysts. J. Power Sources 2015, 286, 166–174. 10.1016/j.jpowsour.2015.03.137.

[ref50] Pu N. W.; Wang C. A.; Liu Y. M.; Sung Y.; Wang D. S.; Ger M. Der. Dispersion of Graphene in Aqueous Solutions with Different Types of Surfactants and the Production of Graphene Films by Spray or Drop Coating. J. Taiwan Inst. Chem. Eng. 2012, 43 (1), 140–146. 10.1016/j.jtice.2011.06.012.

[ref51] Konios D.; Stylianakis M. M.; Stratakis E.; Kymakis E. Dispersion Behaviour of Graphene Oxide and Reduced Graphene Oxide. J. Colloid Interface Sci. 2014, 430, 108–112. 10.1016/j.jcis.2014.05.033. 24998061

[ref52] Liang A.; Jiang X.; Hong X.; Jiang Y.; Shao Z.; Zhu D. Recent Developments Concerning the Dispersion Methods and Mechanisms of Graphene. Coatings 2018, 8 (1), 33 10.3390/coatings8010033.

[ref53] Poorsargol M.; Alimohammadian M.; Sohrabi B.; Dehestani M. Dispersion of Graphene Using Surfactant Mixtures: Experimental and Molecular Dynamics Simulation Studies. Appl. Surf. Sci. 2019, 464 (August 2018), 440–450. 10.1016/j.apsusc.2018.09.042.

[ref54] Du W.; Wu H.; Chen H.; Xu G.; Li C. Graphene Oxide in Aqueous and Nonaqueous Media: Dispersion Behaviour and Solution Chemistry. Carbon 2020, 158, 568–579. 10.1016/j.carbon.2019.11.027.

[ref55] Liu W.; Speranza G. Tuning the Oxygen Content of Reduced Graphene Oxide and Effects on Its Properties. ACS Omega 2021, 6 (9), 6195–6205. 10.1021/acsomega.0c05578. 33718710 PMC7948250

[ref56] Roschger M.; Wolf S.; Genorio B.; Hacker V. Effect of PdNiBi Metal Content : Cost Reduction in Alkaline Direct Ethanol Fuel Cells. Sustainability 2022, 14 (22), 15485 10.3390/su142215485.

[ref57] Nosan M.; Löffler M.; Jerman I.; Kolar M.; Katsounaros I.; Genorio B. Understanding the Oxygen Reduction Reaction Activity of Quasi-1D and 2D N-Doped Heat-Treated Graphene Oxide Catalysts with Inherent Metal Impurities. ACS Appl. Energy Mater. 2021, 4 (4), 3593–3603. 10.1021/acsaem.1c00026.

[ref58] Holdcroft S. Fuel Cell Catalyst Layers: A Polymer Science Perspective. Chem. Mater. 2014, 26 (1), 381–393. 10.1021/cm401445h.

[ref59] Roschger M.; Wolf S.; Mayer K.; Singer M.; Hacker V. Alkaline Direct Ethanol Fuel Cell : Effect of the Anode Flow Field Design and the Setup Parameters on Performance. Energies 2022, 15 (19), 7234 10.3390/en15197234.

[ref60] Hou H.; Wang S.; Jiang Q.; Jin W.; Jiang L.; Sun G. Durability Study of KOH Doped Polybenzimidazole Membrane for Air-Breathing Alkaline Direct Ethanol Fuel Cell. J. Power Sources 2011, 196 (6), 3244–3248. 10.1016/j.jpowsour.2010.11.104.

[ref61] Azadmanjiri J.; Srivastava V. K.; Kumar P.; Wang J.; Yu A. Graphene-Supported 2D Transition Metal Oxide Heterostructures. J. Mater. Chem. A 2018, 6 (28), 13509–13537. 10.1039/C8TA03404D.

[ref62] Åkerlöf G. Dielectric Constants of Some Organic Solvent-Water Mixtures at Various Temperatures. J. Am. Chem. Soc. 1932, 54 (11), 4125–4139. 10.1021/ja01350a001.

[ref63] Park J. H.; Shin M. S.; Park J. S. Effect of Dispersing Solvents for Ionomers on the Performance and Durability of Catalyst Layers in Proton Exchange Membrane Fuel Cells. Electrochim. Acta 2021, 391, 138971 10.1016/j.electacta.2021.138971.

[ref64] Jhong H.-R.; Brushett F. R.; Kenis P. J. A. The Effects of Catalyst Layer Deposition Methodology on Electrode Performance. Adv. Energy Mater. 2013, 3 (5), 589–599. 10.1002/aenm.201200759.

[ref65] Ručigaj A.; Connell J. G.; Dular M.; Genorio B. Influence of the Ultrasound Cavitation Intensity on Reduced Graphene Oxide Functionalization. Ultrason. Sonochem. 2022, 90, 106212 10.1016/j.ultsonch.2022.106212. 36327924 PMC9626748

[ref66] Alzate V.; Fatih K.; Wang H. Effect of Operating Parameters and Anode Diffusion Layer on the Direct Ethanol Fuel Cell Performance. J. Power Sources 2011, 196 (24), 10625–10631. 10.1016/j.jpowsour.2011.08.080.

[ref67] Tian Y.; Yan S.; Song C.; Wang C.; Chen J. Research on the Influence of Micro-Morphology on the Hydrophobicity of Material Surface. Colloids Interface Sci. Commun. 2022, 46, 100556 10.1016/j.colcom.2021.100556.

[ref68] Loukrakpam R.; Gomes B. F.; Kottakkat T.; Roth C. A Bird’s Eye Perspective of the Measurement of Oxygen Reduction Reaction in Gas Diffusion Electrode Half-Cell Set-Ups for Pt Electrocatalysts in Acidic Media. JPhys. Mater. 2021, 4 (4), 044004 10.1088/2515-7639/ac0319.

[ref69] Shypunov I.; Kongi N.; Kozlova J.; Matisen L.; Ritslaid P.; Sammelselg V.; Tammeveski K. Enhanced Oxygen Reduction Reaction Activity with Electrodeposited Ag on Manganese Oxide–Graphene Supported Electrocatalyst. Electrocatalysis 2015, 6 (5), 465–471. 10.1007/s12678-015-0266-x.

[ref70] Yun C.; Hwang S. Analysis of the Charging Current in Cyclic Voltammetry and Supercapacitor’s Galvanostatic Charging Profile Based on a Constant-Phase Element. ACS Omega 2021, 6 (1), 367–373. 10.1021/acsomega.0c04702. 33458488 PMC7807758

[ref71] Connor P.; Schuch J.; Kaiser B.; Jaegermann W. The Determination of Electrochemical Active Surface Area and Specific Capacity Revisited for the System MnOx as an Oxygen Evolution Catalyst. Zeitschrift fur Phys. Chemie 2020, 234 (5), 979–994. 10.1515/zpch-2019-1514.

[ref72] Anantharaj S.; Noda S. How Properly Are We Interpreting the Tafel Lines in Energy Conversion Electrocatalysis?. Mater. Today Energy 2022, 29, 101123 10.1016/j.mtener.2022.101123.

