# Supplementary material for: Influence of the Electrode Deposition Method of Graphene-Based Catalyst Inks for ADEFC on Performance
Source: ACS Appl Mater Interfaces. 2023 Aug 17;15(34):40687–99. doi: 10.1021/acsami.3c09192 (PMC10472432; doi:10.1021/acsami.3c09192)
Supplement: Supplementary file 1 — am3c09192_si_001.pdf [file am3c09192_si_001.pdf]

Supporting Information

Influence of the Electrode Deposition Method of Graphene-based Catalyst Inks for ADEFC on Performance

Michaela Roschger[1,], Sigrid Wolf[1], Richard Hasso[1], Boštjan Genorio[2], Selestina Gorgieva[3], and Viktor Hacker[1]*

[1]*Institute of Chemical Engineering and Environmental Technology, Graz University of Technology, Inffeldgasse 25/C, 8010 Graz, Austria*

[2]*Faculty of Chemistry and Chemical Technology, University of Ljubljana, Večna pot 113, 1000 Ljubljana, Slovenia*

[3]*Faculty of Mechanical Engineering, University of Maribor, Smetanova ulica 17, 2000 Maribor, Slovenia*

Corresponding author:

Michaela Roschger
Graz University of Technology
Institute of Chemical Engineering and Environmental Technology
Inffeldgasse 25/C, 8010 Graz, Austria
E-mail: michaela.roschger@tugraz.at

Table S1: Physical investigation results of the PdNiBi/N-rGO catalyst inks.

Sample	Zeta potential (mV)	Hydr. radius (nm)	Absorbance				
			0 h	1 h	2 h	3 h	6 h
**1**	-43.8 ± 0.9	862	617.0	514.0	432.0	438.0	313.0
**2**	-44.9 ± 1.0	827	611.0	511.0	458.0	392.0	424.0
**3**	-47.8 ± 0.6	1153	634.0	564.0	494.0	484.0	372.0
**4**	-50.6 ± 0.3	1130	572.0	589.0	452.0	464.0	385.0
**5**	-55.6 ± 0.1	863	547.0	535.0	478.0	416.0	250.0
**6**	-53.0 ± 0.6	1043	897.0	866.0	787.0	708.0	573.0
**7**	-52.2 ± 0.3	1034	596.0	436.0	427.0	399.0	266.0
**8**	-53.2 ± 1.0	1346	394.0	243.0	256.0	248.0	141.0
**9**	-55.0 ± 0.3	1845	220.0	129.0	118.0	97.0	92.0
**10**	-50.2 ± 2.3	1590	160.0	87.0	89.0	87.0	75.0
**11**	-39.9 ± 0.9	946	146.0	129.0	119.0	111.0	83.0

Table S2: Physical investigation results of the Ag-Mn_xO_y/N-rGO catalyst inks.

Sample	Zeta potential (mV)	Hydr. radius (nm)	Absorbance				
			0 h	1 h	2 h	3 h	6 h
**1**	-44.0 ± 0.2	1077	645.0	521.0	460.0	433.0	382.0
**2**	-48.0 ± 0.8	729	563.0	505.0	471.0	475.0	406.0
**3**	-33.3 ± 0.2	875	617.0	559.0	509.0	532.0	498.0
**4**	-46.9 ± 1.7	707	542.0	528.0	508.0	421.0	426.0
**5**	-39.3 ± 0.7	1251	628.0	669.0	577.0	548.0	516.0
**6**	-43.7 ± 1.4	1164	716.0	674.0	624.0	612.0	547.0
**7**	-43.5 ± 2.0	1089	683.0	673.0	631.0	577.0	543.0
**8**	-46.4 ± 1.5	806	521.0	464.0	386.0	394.0	347.0
**9**	-46.5 ± 3.6	1340	354.0	269.0	305.0	292.0	264.0
**10**	-40.2 ± 1.1	1020	577.0	527.0	527.0	455.0	424.0
**11**	-44.3 ± 1.0	1251	745.0	684.0	631.0	595.0	503.0

Table S3: EDX results in wt.% of all produced electrodes.

Cathode	C	N	Ag	Mn	O
**spray$_C$**	35.75	14.13	17.95	21.07	11.11
**drop$_C$**	27.83	10.13	21.15	25.89	14.99
**brush$_C$**	36.36	10.55	19.99	16.84	16.25
**roll$_C$**	36.61	14.27	14.12	16.76	18.24

Anode	C	N	Pd	Ni	Bi
**spray$_A$**	37.88	15.61	39.06	3.29	4.16
**drop$_A$**	38.68	16.64	37.58	2.24	4.85
**brush$_A$**	42.74	22.31	28.46	1.75	4.75
**roll$_A$**	38.01	14.01	39.81	3.37	4.80

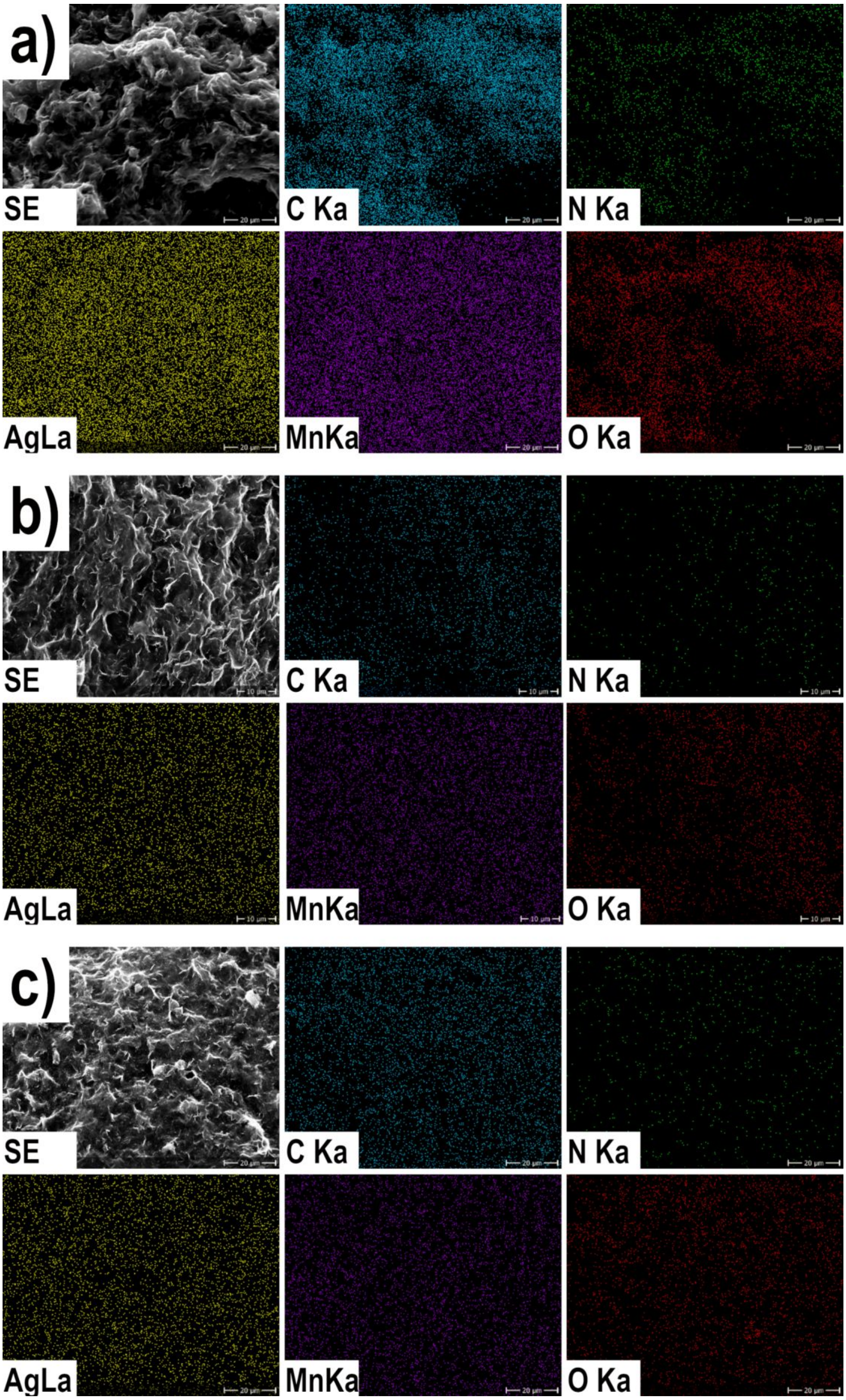

Figure S1: EDX mapping of the produced cathodes a) $spray_C$, b) $drop_C$, and c) $roll_C$.

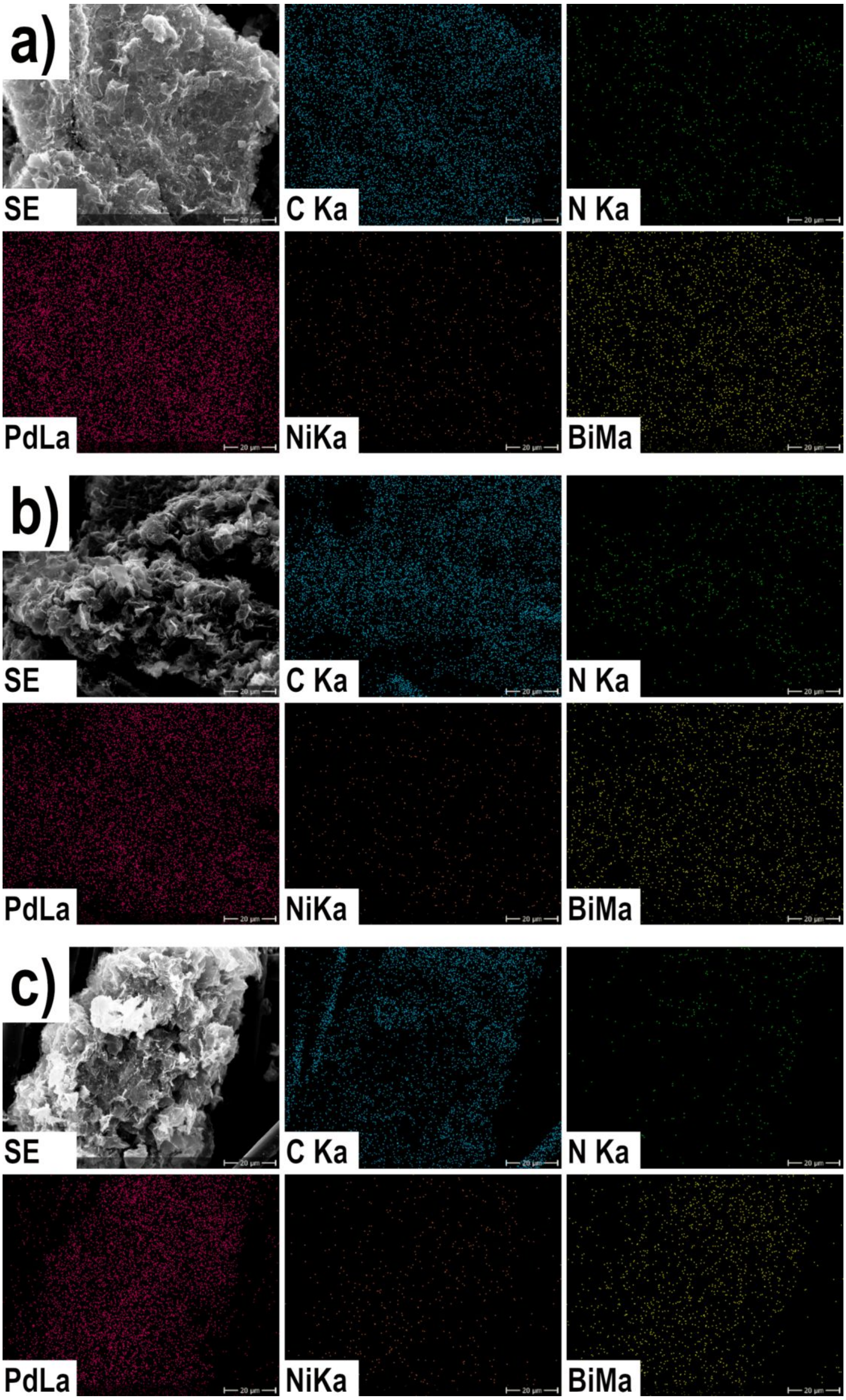

Figure S2: EDX mapping of the produced anodes a) $drop_A$, b) $brush_A$ and c) $roll_A$.

Table S4: Brunauer–Emmett–Teller results and contact angles of all cathodic and anodic electrodes in comparison with the blank gas diffusion layers.

Cathode	S_{BET} (m² g⁻¹)	θ (°)
**blank$_C$**	30.0	160.6 ± 0.4
**spray$_C$**	42.2	152.3 ± 2.0
**drop$_C$**	34.0	146.5 ± 1.7
**brush$_C$**	34.7	155.5 ± 3.5
**roll$_C$**	25.6	139.9 ± 4.8

Anode	S_{BET} (m² g⁻¹)	θ (°)
**blank$_A$**	14.3	142.2 ± 0.8
**spray$_A$**	13.4	145.7 ± 3.2
**drop$_A$**	25.9	136.4 ± 0.8
**brush$_A$**	12.6	146.9 ± 0.4
**roll$_A$**	31.0	143.4 ± 4.3

S_{BET}: BET specific surface area, θ: contact angle

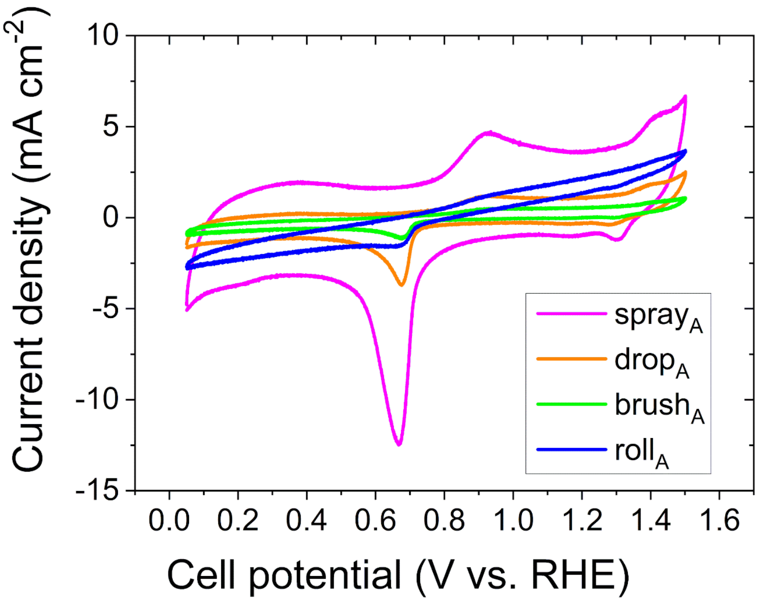

Figure S3: Cyclic voltammogramms of the PdNiBi/N-rGO electrodes with a scan rate of 10 mV s^{-1} in a potential range from 0.05 V – 1.50 V *vs.* RHE in 5 M KOH at RT.

Table S5: Electrochemical half-cell GDE measurement results and Tafel plot values for all cathodic and anodic electrodes.

**Cathode**	**OCP vs. RHE (V)**			**E_{onset}[a] vs. RHE (V)**			**j[b] (mA cm^{-2})**			**|Tafel slope| (|mV dec^{-1}|)**			**|$n\alpha$|**		
	**I**	**II**	**III**	**I**	**II**	**III**	**I**	**II**	**III**	**I**	**II**	**III**	**I**	**II**	**III**
**spray$_C$**	0.900	0.921	0.922	0.843	0.845	0.836	-33	-95	-109	442	186	156	0.13	0.36	0.45
**drop$_C$**	0.892	0.917	0.911	0.854	0.887	0.880	-326	-847	-616	162	142	147	0.36	0.46	0.48
**brush$_C$**	0.892	0.907	0.905	0.853	0.883	0.880	-401	-1075	-951	170	130	137	0.35	0.51	0.51
**roll$_C$**	0.898	0.917	0.918	0.854	0.881	0.881	-277	-594	-592	226	156	151	0.26	0.42	0.47

**Anode**	**OCP vs. RHE (V)**			**E_{onset}[c] vs. RHE (V)**			**j[d] (mA cm^{-2})**			**|Tafel slope| (|mV dec^{-1}|)**			**|$n\alpha$|**		
	**I**	**II**	**III**	**I**	**II**	**III**	**I**	**II**	**III**	**I**	**II**	**III**	**I**	**II**	**III**
**spray$_A$**	0.140	0.064	0.039	0.263	0.142	0.096	935	1268	1356	226	216	206	0.26	0.31	0.34
**drop$_A$**	0.170	0.091	0.058	0.280	0.212	0.144	495	626	595	392	313	275	0.15	0.21	0.26
**brush$_A$**	0.155	0.078	0.051	0.284	0.229	0.112	495	477	635	341	327	280	0.17	0.20	0.25
**roll$_A$**	0.176	0.089	0.053	0.298	0.187	0.124	174	418	654	439	410	294	0.13	0.16	0.24

OCP: open circuit potential; E_{onset}: onset potential; j: current density; α: transfer coefficient; n: number of electrons; [a] at -10 mA cm^{-2}; [b] j at 0.70 V vs RHE; [c] at 10 mA cm^{-2}; [d] j at 0.65 V vs RHE

Table S6: EDX results in wt.% of the electrodes of the N-rGO based MEA after the durability study.

**Cathode**	**C**	**N**	**Ag**	**Mn**	**O**
**brush$_C$**	39.19	14.86	14.78	13.61	17.55

**Anode**	**C**	**N**	**Pd**	**Ni**	**Bi**
**spray$_A$**	31.64	20.29	39.12	2.98	5.98

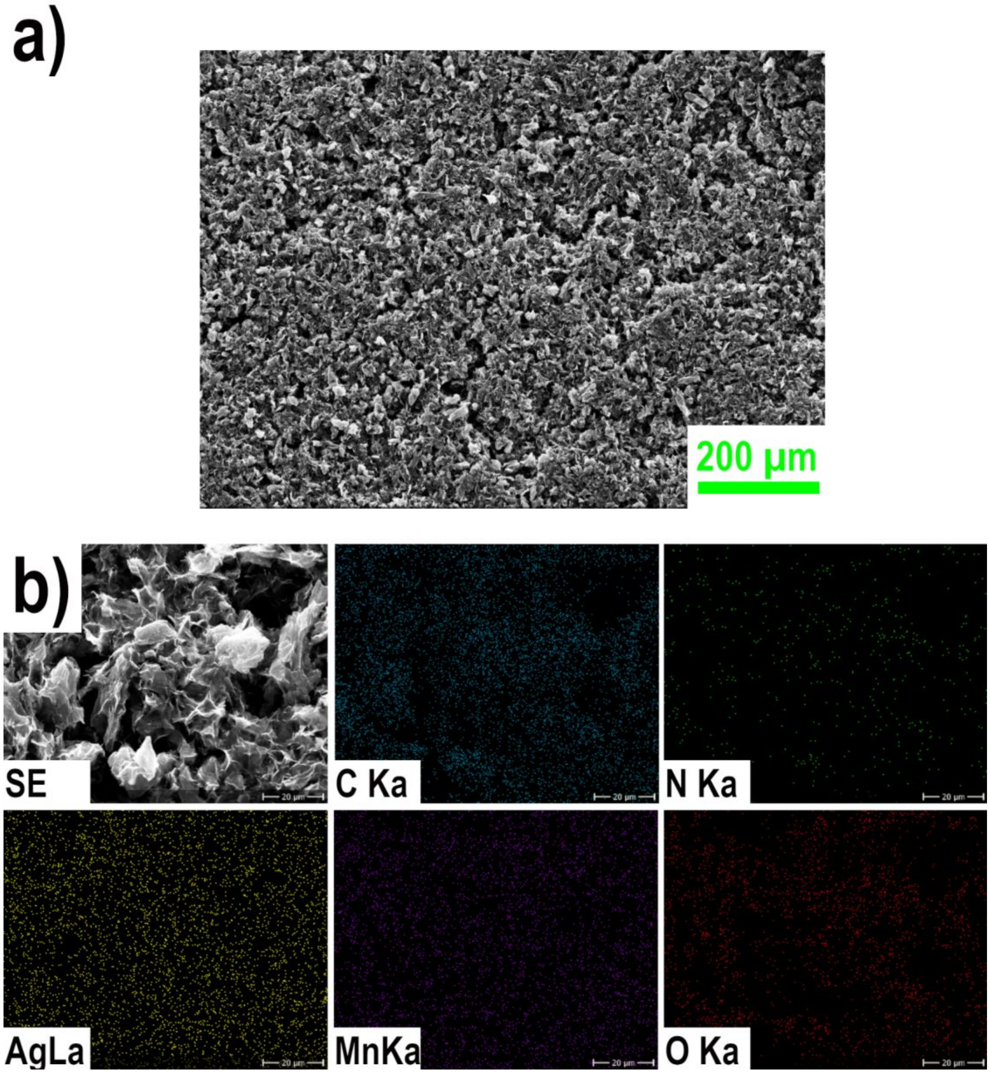

Figure S4: a) SEM images of the electrode coating and b) EDX mapping of the cathodic electrode of the N-rGO based MEA after the durability study.

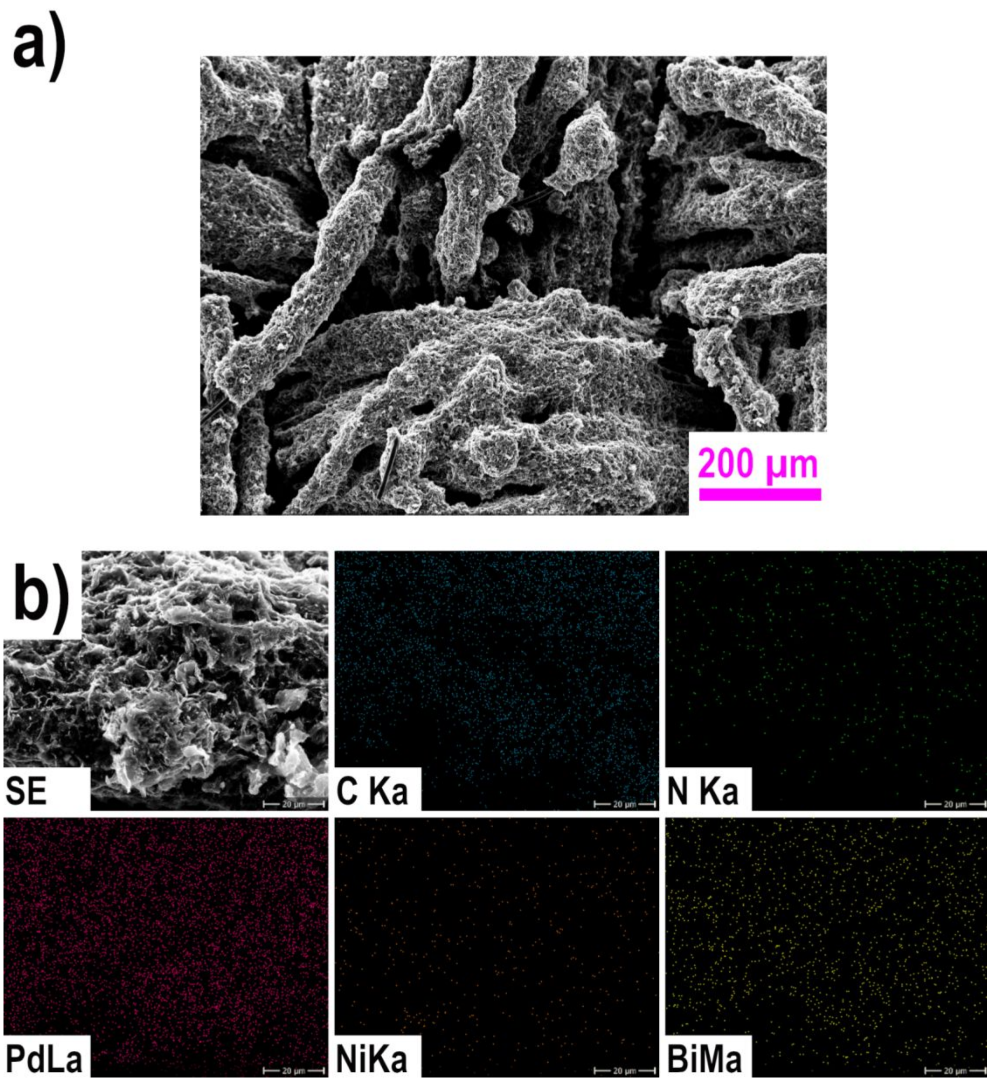

Figure S5: a) SEM images of the electrode coating and b) EDX mapping of the anodic electrode of the N-rGO based MEA after the durability study.
